# Modelling variability in functional brain networks using embeddings

**DOI:** 10.1162/IMAG.a.1188

**Published:** 2026-04-17

**Authors:** Rukuang Huang, Chetan Gohil, Mark Woolrich

**Affiliations:** Oxford Centre for Human Brain Activity (OHBA), Wellcome Centre for Integrative Neuroimaging, Department of Psychiatry, University of Oxford, Oxford, United Kingdom

**Keywords:** functional connectivity, population modelling, unsupervised learning, generative modelling, Bayesian modelling

## Abstract

Functional neuroimaging techniques allow us to estimate functional networks that underlie cognition. However, these functional networks are often estimated at the group level and do not allow for the discovery of, nor benefit from, subpopulation structure in the data, that is, the fact that some recording sessions may be more similar than others. Here, we propose the use of embedding vectors (c.f. word embedding in Natural Language Processing) to explicitly model individual sessions while inferring networks across a group. This vector is effectively a “fingerprint” for each session, which can cluster sessions with similar functional networks together in a learnt embedding space. We apply this approach to estimate dynamic functional networks using a hierarchical Hidden Markov Model (HMM). We call this approach HIVE (HMM with Integrated Variability Estimation). Using simulated data, we show that HIVE can uncover true subpopulation structure and show improved performance over existing approaches. Using real magnetoencephalography data, we show the learnt embedding vectors (session fingerprints) reflect meaningful sources of variation across a population. Overall, HIVE provides a powrful new approach for modelling individual sessions while leveraging information available across an entire group.

## 1 Introduction

*Functional connectivity* (FC), which is defined as the temporal correlation between spatially remote regions (Friston, 1994), is a popular tool used to study neuroimaging data. Many previous FC studies have used time-averaged, or static, estimates of FC to identify functional networks in tasks and at rest (Biswal et al., 1995; Brookes et al., 2011; Filippini et al., 2009; Fox & Raichle, 2007; Honey et al., 2009). However, given the dynamic nature of brain activity (Rabinovich et al., 2012) and the fact that a considerable amount of within-subject variation in FC has been observed (Honey et al., 2009; Meindl et al., 2010; Van Dijk et al., 2010), evidence suggests that FC changes with time and under the influence of task (Esposito et al., 2006; Fornito et al., 2012). Consequently, there is growing interest in the study of dynamic functional networks (Baker et al., 2014; Betti et al., 2013; Brookes et al., 2014; Brovelli et al., 2017; Gohil et al., 2022; Quinn et al., 2018; Vidaurre et al., 2016), which has led to several advancements in the modelling of dynamic FC with methods such as the sliding window approach combined with clustering (Allen et al., 2014; Chang & Glover, 2010), the Hidden Markov Models (the HMMs, Baker et al., 2014; Vidaurre et al., 2016), and Dynamic Network Modes (DyNeMo, Gohil et al., 2022) being proposed.

A key consideration that is often overlooked in the modelling of FC is that there is a considerable amount of inter-subject variability. A common assumption in both static and dynamic FC methods is that the same network, or set of networks, is shared by all subjects, that is, the analysis is done at the group level. This is motivated by the fact that there is limited data per subject. Pooling over as much data as possible (across subjects) improves the estimate of FC by averaging out noise. However, implicitly this means inter-subject variability is considered as noise in the estimate of the group average. Although it is possible to post-hoc estimate the subject-specific FC, for example, dual regression of group independent component analysis (ICA, Beckmann et al., 2009; Nickerson et al., 2017) and dual estimation of the Hidden Markov Model (HMM, Vidaurre et al., 2021), these methods treats each individual independently from other individuals and ignores the relationship with other individuals. Principled hierarchical Bayesian models such as PROFUMO (Farahibozorg et al., 2021; Harrison et al., 2015) improve upon this by jointly inferring group and subject parameters. However, they typically model variability as statistical deviations from a group mean. Our goal is distinct: we aim to explicitly model the latent structure of this variability. By embedding subjects into a low-dimensional space, our approach captures the pairwise relationships and subpopulation structure between individuals, rather than treating them solely as independent samples from a group prior.

Functional neuroimaging data are known to possess significant heterogeneity related to, for instance, intrinsic differences in FC due to demographics (Gohil et al., 2024; Quinn et al., 2024) and systematic differences due to scanner type (e.g., CTF vs Elekta MEG scanners) or site (Yamashita et al., 2019). Disentangling these sources of variability is a key challenge. We wish to identify the particular sources of variability that are of interest, such as the differences in intrinsic FC due to demographics, and remove those that are trivial, such as the scanner type. The intrinsic differences in FC are useful for individualized predictions in downstream tasks such as the development of biomarkers for disease or response to intervention (Fedota & Stein, 2015; Philips et al., 2017; J. J. Taylor et al., 2021; Vidaurre et al., 2021).

Crucially, what is still missing in the current literature is a generative approach that explicitly models the structure of inter-subject variability, rather than treating it solely as statistical deviation from a group average (Farahibozorg et al., 2021; Harrison et al., 2015). While hierarchical models can constrain subject estimates using group priors, they generally do not capture the latent geometric relationships between subjects (such as subpopulations or continuous trends). Our proposed framework in this paper addresses this gap by embedding each subject or session into a shared latent space that captures inter-session/subject similarities and differences. By utilising a hierarchical model to explicitly specify the generative process of individual-level networks from group-level networks, we enable joint inference of individual variability and group-level structure. We describe the framework and its application to the HMM in detail in Section 2 and illustrate its use in Section 3.

## 2 Methods

### 2.1 Previous methods

#### 2.1.1 Hidden Markov modelling

The basic assumption in the HMM is that the brain transitions between states. Each state has different spatiotemporal characteristics and is represented by a multivariate Gaussian distribution. The covariance matrix of the multivariate Gaussian distribution characterises the interaction between different channels/parcels and encodes information about FC. The generative model (SI Section A.1.1) describes how the time series is generated given a set of model parameters (state means, state covariances, and transition probability matrix). Through training, we infer the parameters that best generate the observed data and partition the time series into a finite number of mutually exclusive states.

#### 2.1.2 Dual estimation

When training the HMM, training data are created by concatenating the time series of different sessions. Therefore, the inferred covariance matrix for each state is shared across all sessions. This means the dynamic FC is estimated at the “group level” and the HMM itself does not provide any estimates of individual session networks. Individual estimates of FC can be obtained by using post-hoc analysis of the HMM—a method called *dual estimation* (Vidaurre et al., 2021), which has a similar rationale as dual regression in Independent Component Analysis (ICA, Beckmann et al., 2009; Nickerson et al., 2017). The idea is for each session, we fix the state probabilities from a trained HMM and re-estimate the covariances with the session’s data only. In this paper, we use HMM-DE (HMM with dual estimation) to refer to the pipeline of training a group-level HMM followed by dual estimation. See SI Section A.1.2 for details of performing dual estimation.

It should be noted that due to structures within a population, some sessions may share more similar FC networks than others. However, dual estimation treats each session independently from other sessions and ignores the relationships between different sessions. As a result, it does not allow the inference of dynamic FC networks to pool information across other sessions with similar networks within the group.

#### 2.1.3 Hierarchical models

An alternative to overcome this is to use hierarchical models that capture variability across sessions. In a hierarchical model, parameters are organised into different levels of hierarchy, for example, group and individual level. Individual-level estimates of functional networks are assumed to be generated by a single probabilistic distribution whose parameters are the group-level estimates of functional networks. Hence, the information of functional networks of different sessions is shared through the group level. For example, PROFUMO (Harrison et al., 2015, 2020) has been successfully used to capture aspects of subject variability in functional modes in fMRI data. However, variability within a population is modelled using very simple parametric distributions (e.g., univariate Gaussian), which fails to capture the rich, multivariate variability expected across sessions.

### 2.2 Outline

Here, we extend the HMM to hierarchically model session-specific variability in FC networks. We call this extension HIVE (HMM with integrated variability estimation). Instead of having covariances shared across sessions, we assume that data from different sessions are generated by different sets of covariances, which respects the underlying subpopulation structure, that is, data from similar sessions are generated by similar covariances and vice versa. In what follows, we outline the generative model of HIVE and inference of model parameters. We also describe the datasets studied in this work.

### 2.3 Generative model

**Notations**

- [n]={1,…,n} for n∈N .
- x is a column vector.
- A is a matrix.
- $x_{1:n}=\left\{x_{i}:i\in \left[n\right]\right\}$.

Let ${\text{x}}_{t}^{i}$ be the observed data at time $t\in \left[T_{i}\right]$ for session i∈[N], where N is the number of sessions, $T_{i}$ is the total number of time points of session i, and $x_{t}^{i}$ is a vector of length $N_{c}$—the number of channels/parcels. It is assumed that the data are generated by

$${\text{x}}_{t}^{i}|\left(s_{t}^{i}=j\right)∼N\left(0,\Sigma_{j}^{i}\right)\;\;\;\;\forall t\in \left[T_{i}\right],\forall i\in \left[N\right],$$ (1)

independently. Here, $\Sigma_{j}^{i}$ is the covariance matrix that describes the spatiospectral pattern of session i∈[N] and state j∈[J], where J is the number of states, and the hidden states $s_{1:T_{i}}^{i}$ for each session i∈[N] follow a discrete homogeneous Markov process (Norris, 1998), that is,

$$p\left(s_{1:T_{i}}^{i}\right)=p\left(s_{1}^{i}\right)\prod_{t=2}^{T_{i}}p(s_{t}^{i}|s_{t-1}^{i}),$$ (2)

where $p(s_{t}^{i}=j|s_{t-1}^{i}=j^{\prime })={\text{A}}_{j^{\prime }j}^{i}$ does not depend on time and is the $\left(j^{\prime },j\right)$-th entry to the transition probability matrix ${\text{A}}^{i}$. In this work, we assume that all sessions have the same transition probability matrix ${\text{A}}^{i}=\text{A}\;\forall i\in \left[N\right]$. Next, we hope to model the inter-session relationships between the covariances $\Sigma_{j}^{i}$ and this is achieved by the use of embedding vectors.

#### 2.3.1 Embedding vectors

In this paper, we employ embedding vectors, a technique widely used in the Natural Language Processing literature for characterising semantic relationships between words (Mikolov et al., 2013), to explicitly model between-session variability in the basis set of networks used to describe the functional brain activity.

**Fig. 1.**
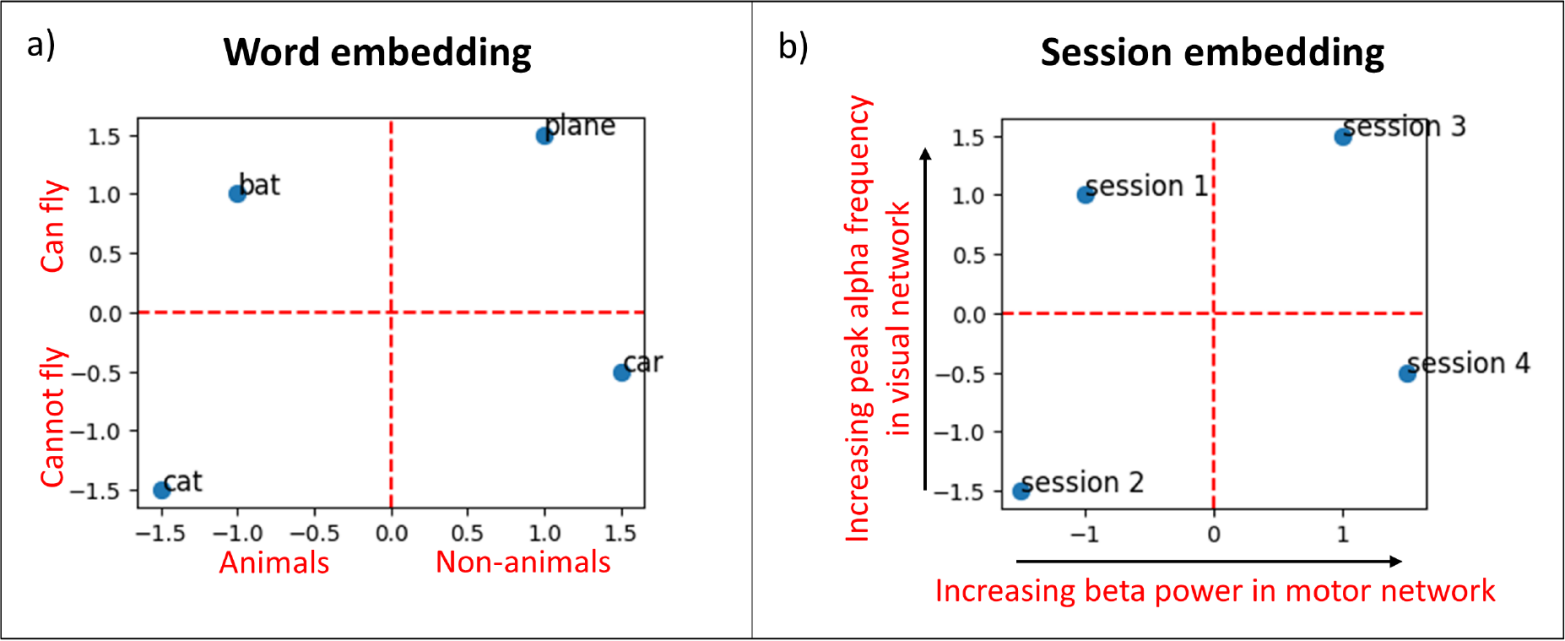
Examples of embedding vectors. (a) Example of word embedding vectors. The x-axis encodes whether a word is for animals or non-animals, and the y-axis encodes whether the object can fly or not. (b) Illustration of possible variability captured by embedding vectors in brain networks found in electrophysiological data such as M/EEG. The x-axis encodes the direction of increasing beta power in motor network, whereas the y-axis encodes the direction of increasing peak alpha frequency in visual network.

A word embedding is a real-valued vector that encodes the meaning of the word such that words closer in the vector space have similar meanings. In Figure 1a, we show an example of word embeddings. Here, the x-axis encodes whether a word is for animals or non-animals and the y-axis encodes whether the object can fly or not. Similarly, we can assign a vector for each recording session and hope that sessions with similar properties are close to each other in the vector space. In Figure 1b, we show an illustration of possible variability captured by embedding vectors. Here, the x-axis encodes the direction of increasing beta power in motor network whereas the y-axis encodes the direction of increasing peak alpha frequency in visual network.

**Fig. 2.**
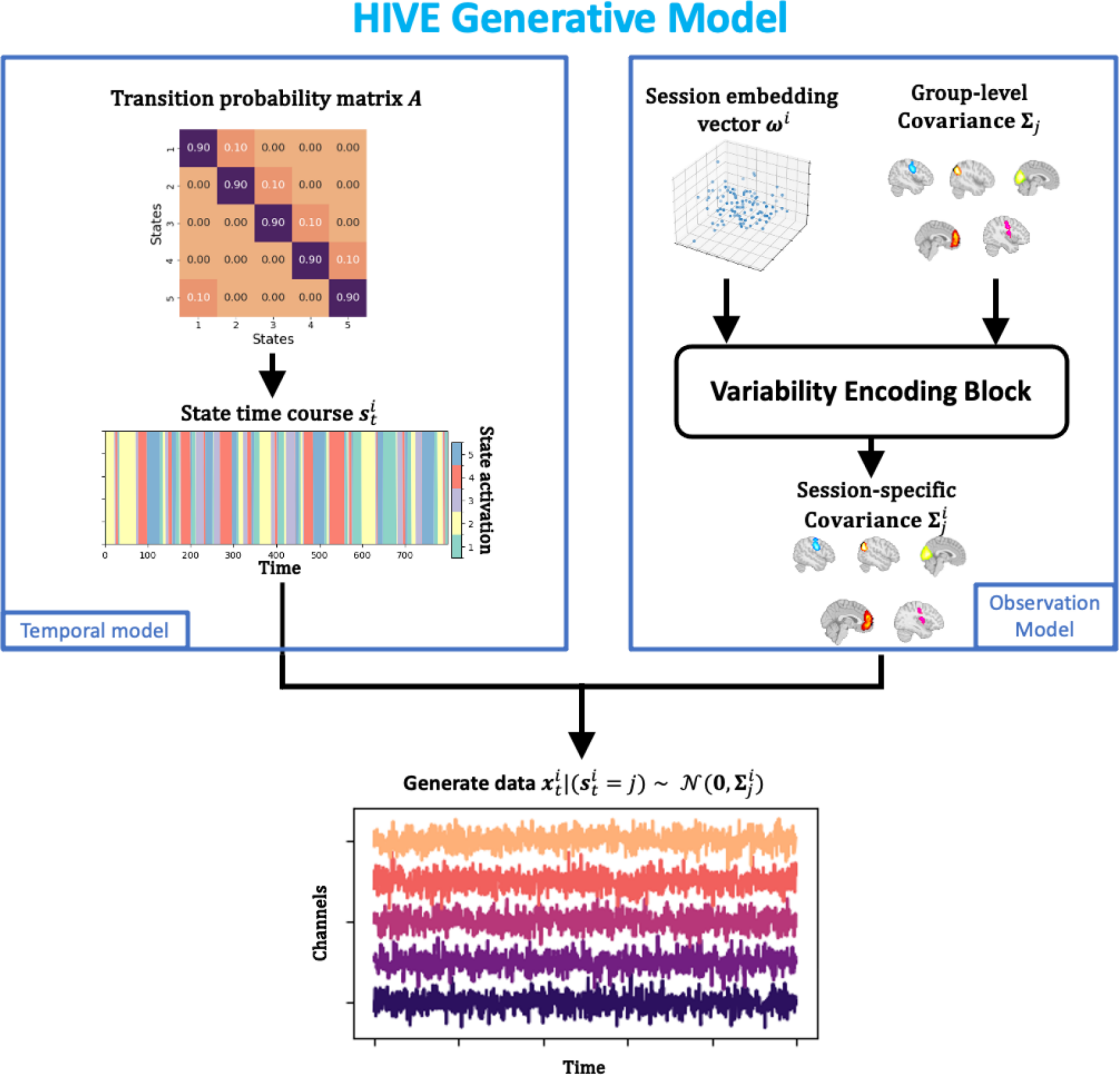
Generative model of HIVE**.** State time courses are generated from the group-level transition probability matrix A through a Markov process. At the same time, session embedding vectors $\omega^{i}$ and group-level covariance $\Sigma_{j}$ are passed to the variability encoding block to give session-specific covariance $\Sigma_{j}^{i}$. Given the state $s_{t}^{i}$ at each time, the observed data are generated through the multivariate Gaussian likelihood.

Embedding vectors have been previously used in computational neuroscience literature to deal with between-subject variability in supervised (Chehab et al., 2021; Csaky et al., 2023) and self-supervised learning (Défossez et al., 2023; Jayalath et al., 2024) contexts. Here, we try to incorporate it into an unsupervised generative model of network activity. Specifically, we propose the *variability encoding block*, which utilises the technique of embedding vectors and hierarchical Bayesian Modelling (Gelman et al., 1995), and apply it to the generative model of the HMM. Conceptually, each session is assigned an embedding vector that acts as a “fingerprint” of this session and contains session-specific information. A function, the variability encoding block, is used to decode the abstract session-specific information hidden in the embedding vectors to the session-specific functional networks. Formally, let $\omega^{i}$ be the embedding vector of session i, a vector of length $n_{\omega }$, where $n_{\omega }$ is called the embedding dimension. Furthermore, let $\Sigma_{j}$ be the group-level covariance matrix of state j. Then, the variability encoding block takes both of $\omega^{i}$ and $\Sigma_{j}$ as inputs and outputs $\Sigma_{j}^{i}$. The generative model of HIVE is summarised in Figure 2.

The generative model describes how data are generated from the model parameters and through model training, and the optimisation process will find the optimal parameters (including embedding vectors) that best describe the data. Ideally, data with similar characteristics are generated from similar embedding vectors and the training process (described in Section 2.4) will group together embedding vectors from similar recording sessions in order to minimise the loss function in Equation (13).

#### 2.3.2 The variability encoding block

In this section, we outline the generative process of session-specific covariances $\Sigma_{j}^{i}$ with the variability encoding block. To ensure legitimate covariance matrices are generated, that is, symmetric and positive definite, we work in the Cholesky space. Let ${\text{L}}_{j},{\text{L}}_{j}^{i}$ be the Cholesky factors (with positive diagonal entries) of $\Sigma_{j},\Sigma_{j}^{i}$ respectively, and furthermore, let ${\text{l}}_{j},{\text{l}}_{j}^{i}$ be the vectors of lower triangular entries of ${\text{L}}_{j},{\text{L}}_{j}^{i}$. Then, there is a bijection between the covariance matrices $\Sigma_{j},\Sigma_{j}^{i}$ and the *Cholesky vectors* ${\text{l}}_{j},{\text{l}}_{j}^{i}$.

The goal of the variability encoding block is to model the session-specific Cholesky vectors ${\text{l}}_{j}^{i}$ as deviations from the group-level Cholesky vectors ${\text{l}}_{j}$, such that the deviations depend on the session-specific information provided by the session embedding vectors $\omega^{i}$ as well as the state-specific information provided by the group-level Choleksy vectors ${\text{l}}_{j}$. Formally, for each session i and each state j, we assume

$${\text{l}}_{j}^{i}={\text{l}}_{j}+d_{j}^{i}{\text{e}}_{j}^{i},$$ (3)

where $d_{j}^{i}$ is a positive scalar representing the magnitude of the deviation and ${\text{e}}_{j}^{i}$ is a standardised vector representing the pattern of deviations. Notice here the group-level Cholesky vectors ${\text{l}}_{j}$ and the session-specific deviations $d_{j}^{i}{\text{e}}_{j}^{i}$ are not identifiable, i.e. there are infinitely many combinations that yield the same session-specific Cholesky vectors ${\text{l}}_{j}^{i}$. Ideally, we prefer the solution where the group-level Cholesky vectors are the “average” of the session-specific Cholesky vectors, that is, we prefer solutions where there is smallest total deviation, while enforcing positivity of this quantity. Hence, we put an exponential prior on $d_{j}^{i}$:

$$d_{j}^{i}∼\text{Exp}\left(\lambda_{j}^{i}\right).$$ (4)

**Fig. 3.**
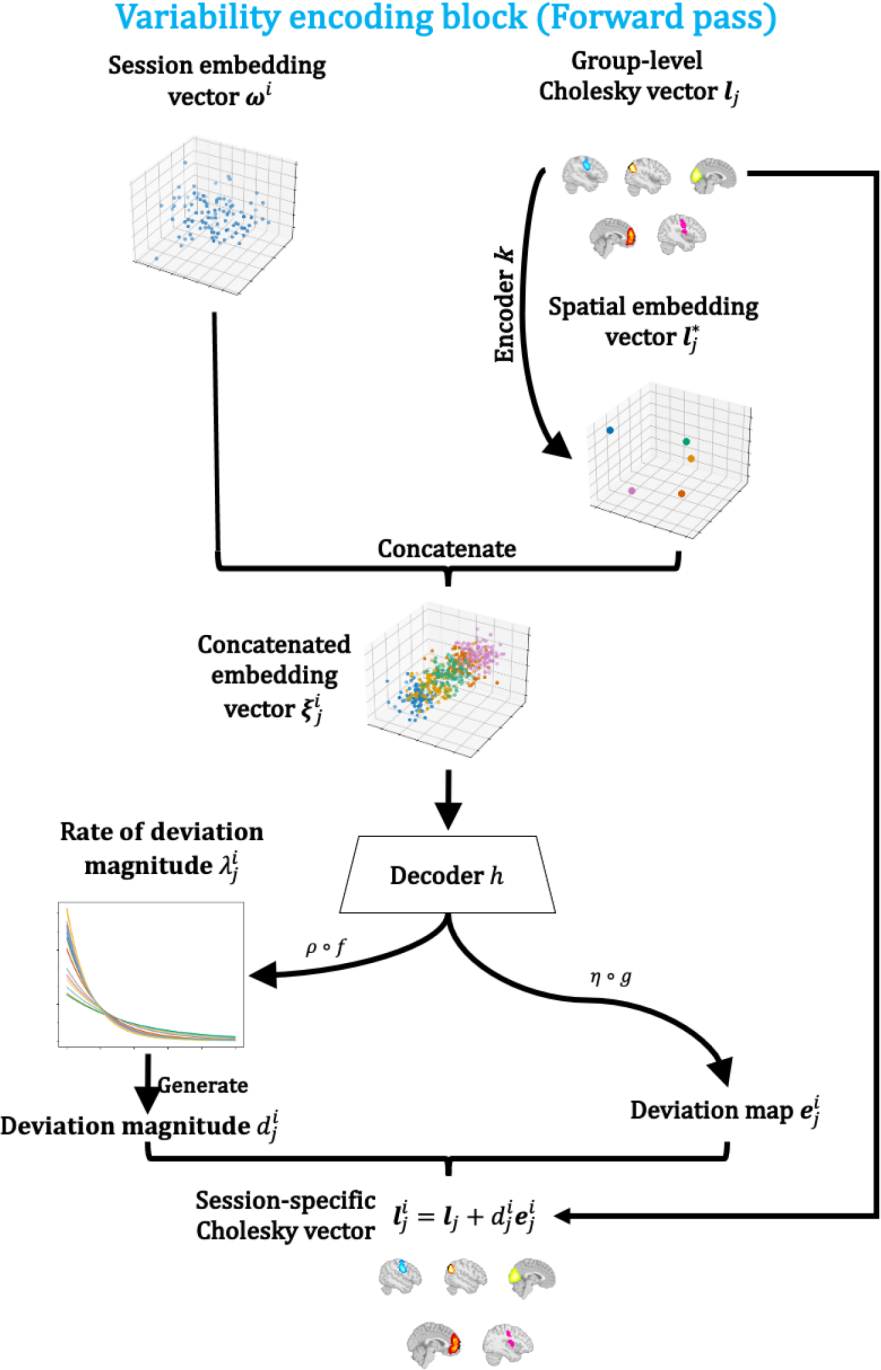
Forward pass of the variability encoding block. Session-specific information from the session embedding vectors $\omega^{i}$ and state-specific information from the group-level Cholesky vectors ${\text{l}}_{j}$ are combined to generate session and state-specific Cholesky vectors ${\text{l}}_{j}^{i}$. Here, the encoder k is an affine transformation that condenses state-specific information to a lower-dimensional space. The decoder h is a multi-layer perceptron (MLP) that decodes state and session-specific deviations from the concatenated embedding vectors $\xi_{j}^{i}$. Deviation magnitude $d_{j}^{i}$ is generated by an exponential distribution with rate $\lambda_{j}^{i}$, which is generated by applying an affine transformation f followed by a softplus ρ activation to the output of h. The deviation map ${\text{e}}_{j}^{i}$ is generated by applying an affine transformation g and normalisation η to the output of h. Finally, we get the session-specific Cholesky vector ${\text{l}}_{j}^{i}$ with ${\text{l}}_{j}^{i}={\text{l}}_{j}+d_{j}^{i}{\text{e}}_{j}^{i}$.

The rate parameter $\lambda_{j}^{i}$ and deviation pattern ${\text{e}}_{j}^{i}$ are generated through

$$\begin{array}{cc} \begin{array}{cc} \lambda_{j}^{i} & =\rho \left(f\left(h\left(\xi_{j}^{i}\right)\right)\right),\\ {\text{e}}_{j}^{i} & =\eta \left(g\left(h\left(\xi_{j}^{i}\right)\right)\right), \end{array} & \end{array}$$ (5)

where ρ is the softplus function to ensure positivity of $\lambda_{j}^{i}$ and η is a Layer Normalisation layer (Ba et al., 2016) with a non-trainable scale parameter of 1 to ensure unit standard deviation of ${\text{e}}_{j}^{i}$. Affine functions f,g are learnable transformations that extract different information from the hidden state given by the decoder h, a multi-layer perceptron (MLP, Popescu et al., 2009). $\xi_{j}^{i}$ is the key object called a concatenated embedding vector that encodes both session and state-specific information. It is formed by concatenating the session embedding vector $\omega^{i}$ and spatial embedding vector ${\text{l}}_{j}^{*}$, which is a lower-dimensional representation of the group-level Cholesky vectors and has length $n_{l^{*}}$:

$$\xi_{j}^{i}=\left[\omega^{i},{\text{l}}_{j}^{*}\right],\;{\text{l}}_{j}^{*}=k\left({\text{l}}_{j}\right),$$ (6)

where k is an affine transformation that serves as an encoder to encode spatial information. As a result, for each session i and each state j, $\xi_{j}^{i}$ is a vector of length $n_{\omega }+n_{l^{*}}$. The complete generative process (forward pass) of the variability encoding block is summarised in Figure 3.

### 2.4 Inference

In this section, we outline the process of inferring the model parameters of HIVE. The model parameters of HIVE, ϕ, include

- the transition probability matrix A,
- the embedding vectors $\omega =\left\{\omega^{i}:i\in \left[N\right]\right\}$,
- the group-level Cholesky vectors $\text{l}=\left\{{\text{l}}_{j}:j\in \left[J\right]\right\}$,
- the weights of the encoder k, the decoder h, the layer normalisation layer η, and the affine transformations f,g .

To perform inference on these parameters, we employ the EM algorithm (Dempster et al., 1977), and more specifically, a variant of the Baum-Welch algorithm (Baum & Eagon, 1967; Baum & Petrie, 1966).

In practice, due to memory restrictions and scalability, it is infeasible to perform inference based on the entire sequence of data. Therefore, data are separated into sequences of length $T_{seq}$ , which is much less than $T=\sum_{i=1}^{N}T_{i}$ - the total number of data points, so that there are $N_{seq}=\left\lfloor T/T_{seq}\right\rfloor$ sequences. For each iteration of the EM algorithm, a random set B of sequences, called a *minibatch*, is used to update the parameters. More specifically, the sequences are split into $\left\lfloor N_{seq}/\left|B\right|\right\rfloor$ minibatches and each minibatch is used in turn for updating the model parameters. After all the minibatches have been processed, called an *epoch*, a different randomised set of minibatches is used for the next epoch.

#### 2.4.1 The EM algorithm

During the “E-step”, we update the state probabilities (the posterior distribution of the state activations) with the forward-backward algorithm of the Baum-Welch algorithm. During the “M-step”, given the state probabilities, we update the transition probability matrix A with the stochastic update technique used in Vidaurre et al. (2018):

$${\text{A}}_{new}=\left(1-\zeta \right){\text{A}}_{old}+\zeta {\text{A}}_{interim},$$ (7)

where ${\text{A}}_{old}$ and ${\text{A}}_{new}$ are the transition probability matrices before and after the update, ${\text{A}}_{interim}$ is the interim update of the transition probability matrix given by the Baum-Welch algorithm, and ζ is the update weight that decreases with training epoch:

$$\zeta \left(epoch\right)={\left(epoch+delay\right)}^{-forget},$$ (8)

where delay=5 and forget=0.7 in this work. Next, we need to infer the rest of the parameters in ϕ and notice we do not have access to the exact posterior distribution of the deviation magnitudes p(d|x) , where $\text{d}=\left\{{\text{d}}^{i}:i\in \left[N\right]\right\}$ $=\left\{d_{j}^{i}:i\in \left[N\right],j\in \left[J\right]\right\}$ and $\text{x}=\left\{{\text{x}}_{1:T_{i}}:i\in \left[N\right]\right\}$ due to the fact that the prior distribution p(d) is parameterised by a complex neural network. Therefore, we approximate p(d|x) using the variational distribution q(d) with a mean field approximation (Blei et al., 2017):

$$q\left(\text{d}\right)=\prod_{i=1}^{N}\prod_{j=1}^{J}q\left(d_{j}^{i}\right)=\prod_{i=1}^{N}\prod_{j=1}^{J}\Gamma (d_{j}^{i}|a_{j}^{i},b_{j}^{i}).$$ (9)

Here, the shape $a_{j}^{i}$ and the rate $b_{j}^{i}$ parameters of the Gamma distributions Γ are learnable parameters. The reason that Gamma distributions are chosen is that there is an analytic solution for the KL divergence between a Gamma distribution and an Exponential distribution. To sum up, the model parameters ϕ, except the transition probability matrix A, and the variational parameters $\psi =\left\{a_{j}^{i},b_{j}^{i}:i\in \left[N\right],j\in \left[J\right]\right\}$ are inferred jointly by minimising the variational free energy given the state probabilities.

#### 2.4.2 Variational free energy given the state probabilities

While breaking the data into sequences, we ensure that data in each sequence n belong to the same session $i_{n}$. Let ${\text{x}}_{1:T_{seq}}^{\left(n\right)}$ be the n-th sequence in the data and similarly for $s_{1:T_{seq}}^{\left(n\right)}$, then the loss function for optimisation for sequence n is the usual variational free energy (Kingma & Welling, 2013):

$$\begin{array}{l} {ℒ}_{n}(\phi ,\psi )=-LL_{n}(\phi ,\psi )+KL_{n}(\phi ,\psi )\\ \;\;\;\;\;\;\;\;\;\;\;\;\;\;\;\;\;\;\;=\underset{LL_{n}(\phi ,\psi )}{\underset{︸}{-E_{q(s_{1:T_{seq}}^{\left(n\right)})}E_{q\psi (d^{i_{n}})}[\log p_{\phi }(x_{1:T_{seq}}^{\left(n\right)},d^{i_{n}})}}+\underset{KL_{n}(\phi ,\psi )}{\underset{︸}{\frac{1}{Nseq}D_{KL}(q_{\psi }(d)||p_{\phi }(d))}}, \end{array}$$ (10)

where $q\left(s_{1:T_{seq}}^{\left(n\right)}\right)$ are the posterior probabilities given by the forward-backward procedure in the Baum-Welch algorithm, $D_{KL}$ is the Kullback-Leibler divergence (Kullback & Leibler, 1951), and the $\frac{1}{N_{seq}}$ factor is due to the fact that the loss is averaged over sequences in a batch. The ${\text{LL}}_{n}\left(\phi ,\psi \right)$ term acts as a reconstruction loss that describes how well the data are reconstructed by the parameters ϕ,ψ and the ${\text{KL}}_{n}\left(\phi ,\psi \right)$ term acts as a regularisation term that penalises complex variational distributions $q_{\psi }\left(\text{d}\right)$ that deviates from the prior $p_{\phi }\left(\text{d}\right)$. The ${\text{LL}}_{n}\left(\phi ,\psi \right)$ term can be simplified as

$$\begin{array}{l} {\text{LL}}_{n}\left(\phi ,\psi \right)=\sum_{t=1}^{T_{seq}}\sum_{j=1}^{J}\mathbb{P}(s_{t}^{\left(n\right)}=j|{\text{x}}_{1:T_{seq}}^{\left(n\right)})\\ \;\;\;\;\;\;\;\;\;\;\;\;\;\;\;\;\;\;E_{q_{\psi }({\text{d}}^{i_{n}})}\left[\text{log }N\;({\text{x}}_{t}^{\left(n\right)}|0,\Sigma_{j}^{i_{n}}\left(\phi ,d_{j}^{i_{n}}\left(\phi \right)\right))\right], \end{array}$$ (11)

where $\mathbb{P}(s_{t}^{\left(n\right)}=j|{\text{x}}_{1:T_{seq}}^{\left(n\right)})$ is the posterior probability that the state at time t of sequence n is j. The ${\text{KL}}_{n}\left(\phi ,\psi \right)$ term can be simplified as

$${\text{KL}}_{n}\left(\phi ,\psi \right)=\frac{1}{N_{seq}}\sum_{i=1}^{N}\sum_{j=1}^{J}D_{KL}\left(\Gamma \left(a_{j}^{i},b_{j}^{i}\right)||\text{Exp}\left(\lambda_{j}^{i}\left(\phi \right)\right)\right).$$ (12)

In summary, for a randomly drawn minibatch $B\subseteq \left[N_{seq}\right]$, the training involves getting the posterior probabilities of the states with the forward-backward procedure, updating the transition probability matrix A with the stochastic update technique, and one step of gradient descent type update (in this paper, the ADAM optimiser is used, Kingma & Ba, 2014) with the loss function

$$ℒ\left(\phi ,\psi \right)=\frac{1}{\left|B\right|}\sum_{n\in B}{ℒ}_{n}\left(\phi ,\psi \right),$$ (13)

with respect to the parameters ϕ,ψ . As aforementioned, we have the exact formula for the Kullback-Leibler divergence between a Gamma and an Exponential distribution, so that the gradient of ${\text{KL}}_{n}\left(\phi ,\psi \right)$ with respect to ϕ,ψ can be calculated. However, the same cannot be said for the ${\text{LL}}_{n}\left(\phi ,\psi \right)$ term which involves an integral with respect to the variational distribution $q_{\psi }\left({\text{d}}^{i_{n}}\right)$. Luckily, we can get an estimate of the gradient with the help of the reparameterisation trick (Kingma & Welling, 2013) for Gamma distributions (Figurnov et al., 2018). The whole training pipeline is implemented in the *osl-dynamics* toolbox (Gohil et al., 2023) with the *Tensorflow* package, which allows trivial calculation of gradients with respect to all the parameters and easy execution of back-propagation. A flowchart summarising the loss calculation is shown in SI section A.1.3 (Fig. A1).

#### 2.4.3 Initialisation of variational parameters

A good initialisation is paramount in training deep neural networks to avoid slow and unstable training. In practice, we found that a good initialisation of the variational parameters ψ is crucial for the training of HIVE, without which the model can either get stuck in local minimum or diverge. We initialise the shape and rate parameters of the Gamma distributions as follows:

$$\begin{array}{c} d_{mean}=\frac{1}{N}\sum_{i=1}^{N}\left|l_{static}^{i}-\bar{l_{static}^{i}}\right|\\ d_{var}=\frac{1}{5}d_{mean}\\ a_{j}^{i}=\frac{d_{mean}^{2}}{d_{var}}\;\;\;\forall i\in \left[N\right],j\in \left[J\right]\\ b_{j}^{i}=\frac{d_{mean}}{d_{var}}\;\;\;\;\;\forall i\in \left[N\right],j\in \left[J\right]. \end{array}$$ (14)

The idea is to estimate the mean deviation magnitude $d_{mean}$ with the deviation of the Cholesky vectors ${\left\{l_{static}^{i}\right\}}_{i=1}^{N}$ of the static covariance matrices from different sessions and get the parameters so that the resulting Gamma distribution has mean $d_{mean}$ and variance $d_{var}=\frac{1}{5}d_{mean}$ , which is what we found to be a good initialisation in practice.

#### 2.4.4 Annealing during training

During training, we employed two annealing techniques. The first one is KL annealing (Bowman et al., 2015) where we start the training without the KL term in the loss and gradually increase the contribution of the KL term. We refer the readers to SI Section A.1.4 for more details.

The second one is that we anneal the sampling process of the variational distribution of deviation magnitudes q(d) when applying the reparameterisation trick:

$$\hat{\text{d}}=\left(1-\kappa \right)\bar{\text{d}}+\kappa \;{\text{d}}^{*},$$ (15)

where $\bar{\text{d}}$ is the expectation of q(d), ${\text{d}}^{*}$ is a sample from q(d), and κ is the annealing factor that is zero at the beginning of the training and gradually increases to one. We find this significantly improves the convergence of the model and we believe this serves as an exploration-exploitation mechanism, which is extensively studied in the fields of reinforcement learning (Wang et al., 2018) and Bayesian Optimisation (Jalali et al., 2012). At the beginning of training, only the expectation is used and the gradient will intend to push the mean deviations to the correct position. This is the exploration phase. As training progresses, we gradually take into account the variance and higher moments of the variational distribution, which allows the training to fine tune the variational parameters. This is the exploitation phase.

### 2.5 Choosing the embedding dimension

We need to pre-define the length $n_{\omega }$ of the embedding vectors $\omega^{i}$. A smaller $n_{\omega }$ will lead to a more parsimonious model but there could be a risk of over-regularisation. On the other hand, a larger $n_{\omega }$ will lead to a more flexible model but could lead to overfitting. In practice, we use the loss function and follow the following procedure. Firstly, we define a set of candidate values for $n_{\omega }$, say $\left\{n_{\omega }^{1},\ldots ,n_{\omega }^{M}\right\}$ where $n_{\omega }^{k}<n_{\omega }^{k+1},\forall k\in \left[M-1\right]$. Then, we train the model with each of these candidates of embedding dimension a number of times independently and for each run, we get the training loss (variational free energy on the training data) of the model. Starting from k=1 , we test whether the training loss of the model with embedding dimension $n_{\omega }^{k+1}$ is significantly lower than that with embedding dimension $n_{\omega }^{k}$. If it is, we continue to test for $n_{\omega }^{k+1}$ and $n_{\omega }^{k+2}$ . If it is not, we choose $n_{\omega }^{k}$ as the embedding dimension. The test for significance can be done with a one sided t-test or a non-parametric permutation test. Formally, the procedure is summarised in Algorithm 1.

**Table:**
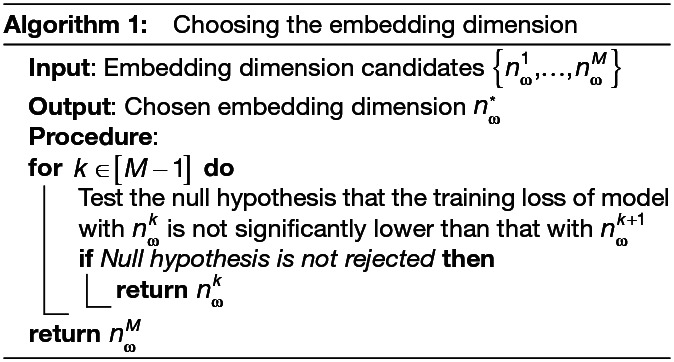


### 2.6 Datasets

#### 2.6.1 Simulated datasets

In the simulation studies, we simulate data with an HMM generative model. To do this, we specify a transition probability matrix and randomly simulate covariance matrices for each state (sessions can have different covariances). Subsequently, a Markov chain is simulated with the pre-specified transition probability matrix. Lastly, at each time point, data are simulated using a multivariate Gaussian distribution with the state covariance matrix which is active at the given time. Gaussian noise is also added to the final time series data (see SI Section A.1.5 for details). The simulated data for the 3 simulation studies are described below.

- **Simulation 1**. We simulate data with $N=10,\;\;T_{i}=25,600,\;\;\forall i\in \left[N\right],\;\;N_{c}=11,\;\;J=5$ . Here, the state covariance matrices of sessions 1 and 2 are altered and all other sessions have the same unaltered group-level covariances—session 1 has increased variance in channel 1 and decreased variance in channel 2 while session 2 has decreased variance in channel 1 and increased variance in channel 2. For both session 1 and 2, the increased variances are 5 times and the decreased variances are 20% of the group-level variance.
- **Simulation 2**. Here, $N=100,\;\;T_{i}=3,000,\;\;\forall i\in \left[N\right],$ $N_{c}=40,\;\;J=5$ . Sessions are assigned into 3 groups, and an embedding vector for each session is simulated according to the session’s assigned group. Session-specific covariances are simulated based on the simulated embedding vectors (see SI Section A.1.5.3 for details). Notice, this is a generalisation of the data in Simulation 1, where one group contains 8 sessions and each of the other 2 groups contain only 1 session, that is, there is no variance within these groups.
- **Simulation 3**. In this study, 100 datasets are simulated, where 10 datasets are simulated for each N∈{10k:k=1,…10} and $T_{i}=3000,\;\forall i\in \left[N\right],$ $N_{c}\;=40,\;\;J=5$ . Session-specific covariances are simulated in the same way as in Simulation 2.

#### 2.6.2 Real MEG data

We demonstrate the use cases of the proposed model with 3 publicly available MEG datasets, including two resting-state and one visual task dataset. The datasets are source-reconstructed and parcellated to 38 regions of interest. We describe the steps of data processing before model training below.

- **Raw data**. The first resting-state dataset (J. R. Taylor et al., 2017, we refer to this dataset as the Cam-CAN dataset) contains eyes-closed data from 612 healthy participants. These data were collected using an Elekta Neuromag Vectorview 306 scanner at a sampling frequency of 1 kHz. A highpass filter of 0.03 Hz and MaxFilter were applied. In the visual task MEG dataset (Wakeman & Henson, 2015, we refer to this dataset as the Wakeman-Henson dataset), each of the 19 health participants were scanned 6 times, during which 3 types of visual stimuli were shown to the participants. The data were also collected using an Elekta Neuromag Vectorview 306 scanner. The second resting-state dataset was collected using a 275-channel CTF scanner. This dataset (we refer to this dataset as the Nottingham dataset) contains eyes-closed data from 64 healthy participants, collected at Nottingham University, UK as part of the MEGUK partnership.
- **Preprocessing**. The Cam-CAN and Nottingham datasets were preprocessed with the same pipeline using the *osl-ephys* package (van Es et al., 2024). The data were band-pass filtered between 0.5 Hz and 125 Hz to remove high-frequency noise and low-frequency drifts. This is followed by a notch filter at 50 Hz and 100 Hz to remove a known artefact due to power line. Then, the data were downsampled to 250 Hz to reduce computational load. Additionally, automated bad segment and bad channel detection, with the generalised ESD procedure (Rosner, 1983), were applied to remove abnormally noisy segments and channels of the recording. Finally, an independent component analysis (ICA) with 64 components was applied. Components with high correlation (0.35) with ECG and EoG recordings are removed. The preprocessing of the Wakeman-Henson dataset is the same as above except that in the end, ICA with 40 components was applied.
- **Source reconstruction and parcellation.** Coregistration and source reconstruction were done using OSL. Structural data were coregistered with the MEG data using an iterative close-point algorithm and digitised head points acquired with a Polhemous pen were matched to individual subject’s scalp surfaces extracted with FSL’s BET tool (Jenkinson et al., 2005; Smith, 2002). The nose was not included in the coregistration as the structural MRI images were defaced. Preprocessed sensor data were source reconstructed onto an 8 mm isotropic grid using a linearly constrained minimum variance beamformer (Van Veen & Buckley, 1988). Voxels were then parcellated into 38 anatomically defined regions of interest, before the symmetric spatial leakage correction described in Colclough et al. (2015) was applied.
- **Data preparation.** Before model training, we follow the preparation steps described in Gohil et al. (2022). The data were time-delay embedded with ±7 lags. Then, principal component analysis (PCA) was used to reduce the dimensionality to 80 channels before a standardisation step (z-transform) was applied to make sure each channel has zero mean and variance of one.

## 3 Results

In this section, we validate HIVE, show its use cases, and compare to HMM-DE on both simulated and real data. We have included model specifications and details on hyperparameters for the models trained on different datasets in SI Section A.1.6. Scripts for reproducing the results are also publicly available on github.com/OHBA-analysis/Huang2025_ModelVariabilityWithEmbeddings.

### 3.1 Simulation

Here, we show the results of training HIVE on the 3 simulated datasets described in Section 2.6.1 and compare these results with those from HMM-DE. We wish to highlight the advantages of HIVE in the following aspects:

- We wish to demonstrate HIVE embeddings reflect inter-subject variability and similarity: Simulation 1 and 2 do this.
- We wish to demonstrate correct state inference with a known ground truth: Simulation 2 does this.
- We wish to demonstrate HIVE infers better individualised networks (state covariances) compared to existing methods (HMM-DE): Simulation 3 does this.

#### 3.1.1 Simulation 1: Variability encoding block learns session-specific covariance deviations

**Fig. 4.**
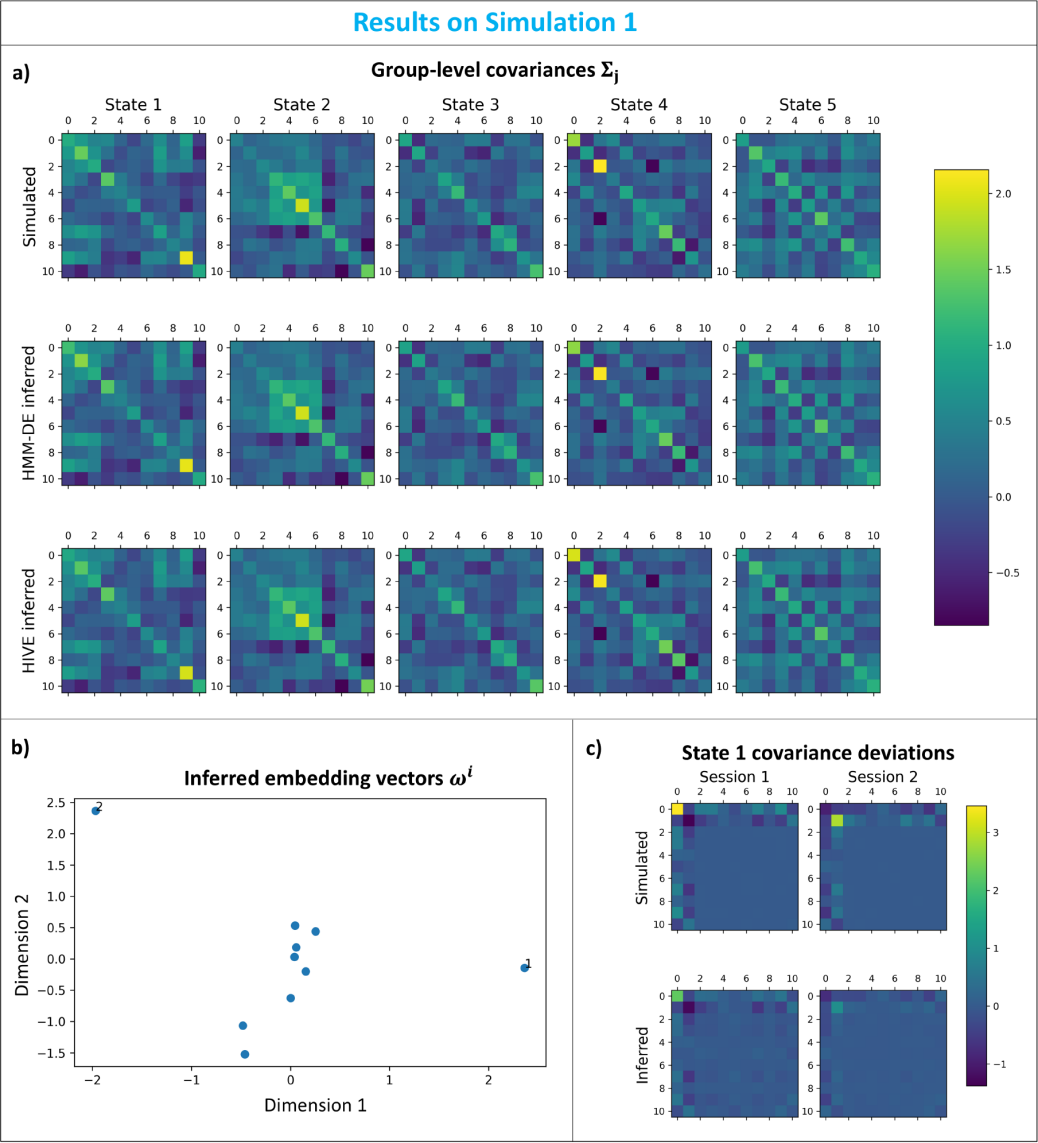
Simulation 1: Variability encoding block learns session-specific covariance deviations. (a) Simulated (top row), HMM-DE inferred (middle row), and HIVE inferred (bottom row) group-level covariances. The columns show covariances of different states. (b) Inferred session embedding vectors. Sessions 1 and 2 are annotated. (c) Simulated deviations (top row) and deviations from trained generative model of HIVE (bottom row) for state 1 for session 1 (left column) and for session 2 (right column).

We aim to test if patterns of deviations across multiple channels can be learnt by the generative model, in particular, the variability encoding block, during inference. This is an important feature in the sense that we want the generative model to act as a prior that regularises how each session can deviate from the group. Firstly, we can see from Figure 4a that both HMM-DE and HIVE can infer the group-level covariances accurately.

What HIVE offers in addition is shown in Figure 4b where the inferred session embedding vectors are plotted. We can clearly see that the embedding vectors for sessions 1 and 2 are far away from the cluster formed by other sessions which have unaltered covariances, which matches well with the ground truth. The model’s capability to encode deviation pattern across multiple channels in the generative model is illustrated in Figure 4c, in which the simulated patterns of deviation for sessions 1 and 2 can be generated from the trained variability encoding block. Although HMM-DE can infer session-specific covariances via dual estimation, it cannot generate data with patterns that deviate from the group-level average. This is because intrinsically HMM-DE is a group-level model.

#### 3.1.2 Simulation 2: HIVE correctly infers the similarity between sessions

**Fig. 5.**
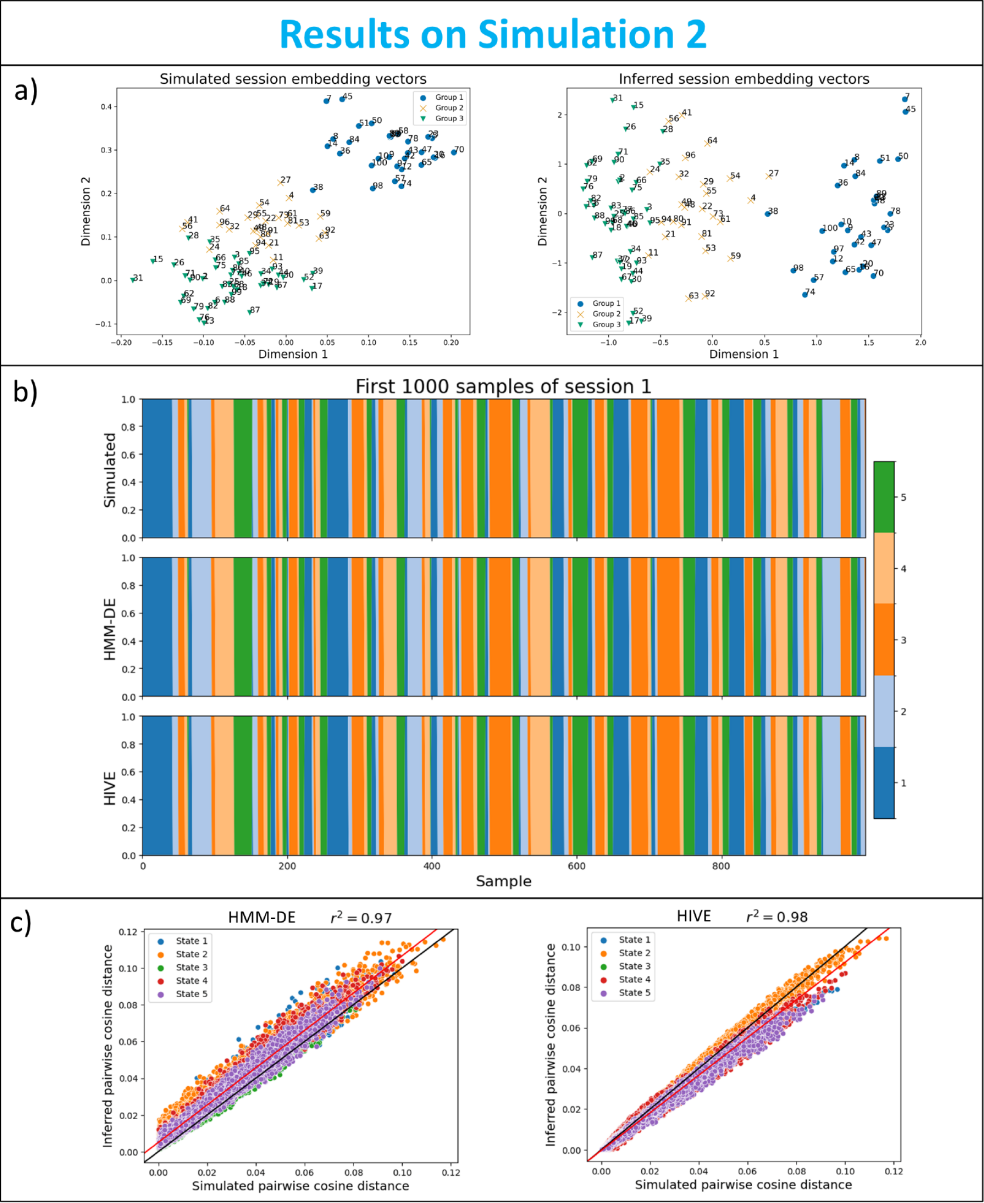
Simulation 2: HIVE correctly infers the similarity between sessions. (a) Simulated (left) and LDA-projected inferred (right) session embedding vectors. Each point is marked and coloured by the ground-truth group assignment, and is annotated by the session number. (b) Simulated (top), HMM-DE inferred (middle), and HIVE inferred (bottom) state time courses. (c) Session-pairwise cosine distance of simulated (x-axis) against inferred (y-axis) covariances from HMM-DE (left) and HIVE (right). The black line shows the y=x line, which corresponds to optimal performance, and the red line is a fitted line through the points, with the coefficient of determinant $r^{2}$ displayed in the title.

In this simulation study, we aim to show that HIVE can recover the ground truth underlying subpopulation structure (simulated embedding vectors of sessions). This is demonstrated in Figure 5a, where the ground-truth grouping of sessions is recovered by the inferred session embedding vectors. In particular, sessions which are close together in the simulated space (e.g., sessions 7 and 45) stay close in the inferred space. Figure 5b shows both HMM-DE and HIVE can infer the state time courses perfectly. Furthermore, we can see from Figure 5c that both approaches can recover the pairwise session relationship between session-specific covariances, though HMM-DE overestimates the pairwise distance due to noise added to the data, whereas HIVE has the preferable behaviour of underestimating the pairwise distance due to the regularising effect of the prior on the deviation magnitude. This prior makes the inferred session-specific covariances more similar to the group average, when there is insufficient evidence available in the data to do otherwise.

#### 3.1.3 Simulation 3: HIVE improves the estimation of session variability

**Fig. 6.**
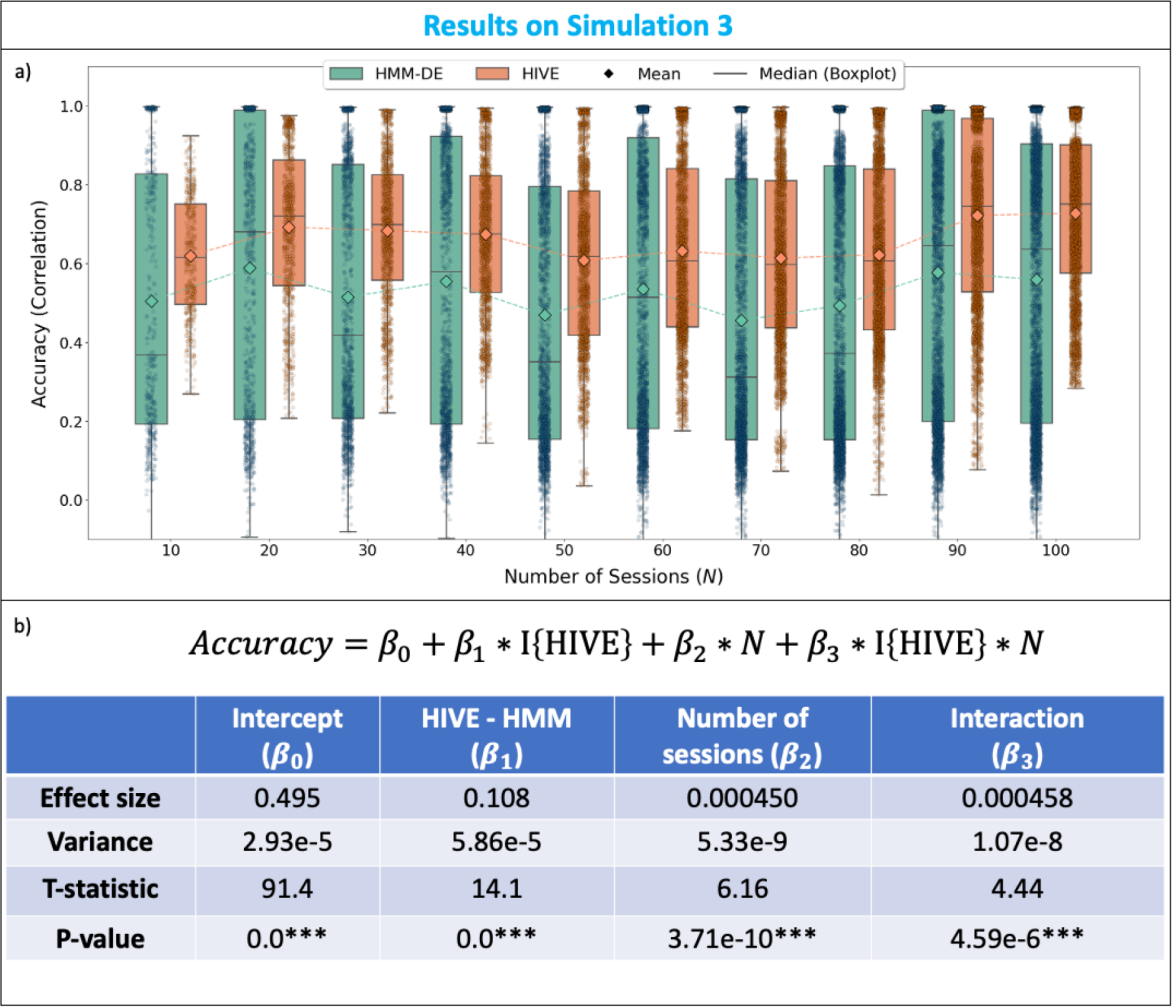
Simulation 3: HIVE improves the estimation of session variability. (a) Accuracy (correlation with ground-truth session-specific covariances) is plotted against the number of sessions in the dataset. Each dot corresponds to the accuracy of inferred session-specific covariances for each state and each session. (b) A regression is fitted to explore advantages of HIVE over HMM-DE. Values are shown in 3 significant figures.

Now, we focus on comparing HIVE and HMM-DE in terms of accuracy of inferred session-specific covariances. Both HIVE and HMM-DE are trained on each of the 100 simulated datasets. In Figure 6a, the accuracy (correlation with ground-truth session-specific covariances) of the inferred session-specific covariance for each state and each session is plotted against the number of sessions in the dataset. We can see qualitatively HIVE achieves better performance compared to HMM-DE. In particular, HIVE always achieves higher mean and median accuracy than HMM-DE across all number of sessions N. Notably, we can observe that a significant mass of the accuracies from HMM-DE is concentrated at low values (<0.3 ), whereas there are less extremely low accuracies from HIVE. This again shows HIVE is more robust to noise in the data.

In order to more rigorously investigate the advantages of HIVE over HMM-DE, we regress the accuracies on whether HIVE is used, the number of sessions (N), and the interaction between the two:

$$\begin{array}{l} Accuracy=\beta_{0}+\beta_{2}\times 1\left\{\text{HIVE}\right\}+\beta_{2}\times N+\beta_{3}\\ \;\;\;\;\;\;\;\;\;\;\;\;\;\;\;\;\;\;\times 1\left\{\text{HIVE}\right\}\times N. \end{array}$$ (16)

The results of this fitted regression are summarised in Figure 6b. In particular, the baseline accuracy of HIVE is 0.108 higher than HMM-DE and this effect is significant. Furthermore, the accuracy of HMM-DE increases by 0.00045 per number of session, which translates to 0.0450 per increase of 100 sessions, and this effect is also significant. More importantly, the interaction term $\beta_{3}$ has a significantly positive effect, meaning for each increase of 100 sessions, the increase in accuracy when using HIVE is 0.0458 higher than using HMM-DE (i.e., approximately doubled the increase in accuracy). This shows the advantage of the variability encoding block in making use of data of heterogeneous sessions to help infer on every single session.

### 3.2 Real MEG data

#### 3.2.1 Wakeman Henson: HIVE reveals similarities and differences between MEG recordings

**Fig. 7.**
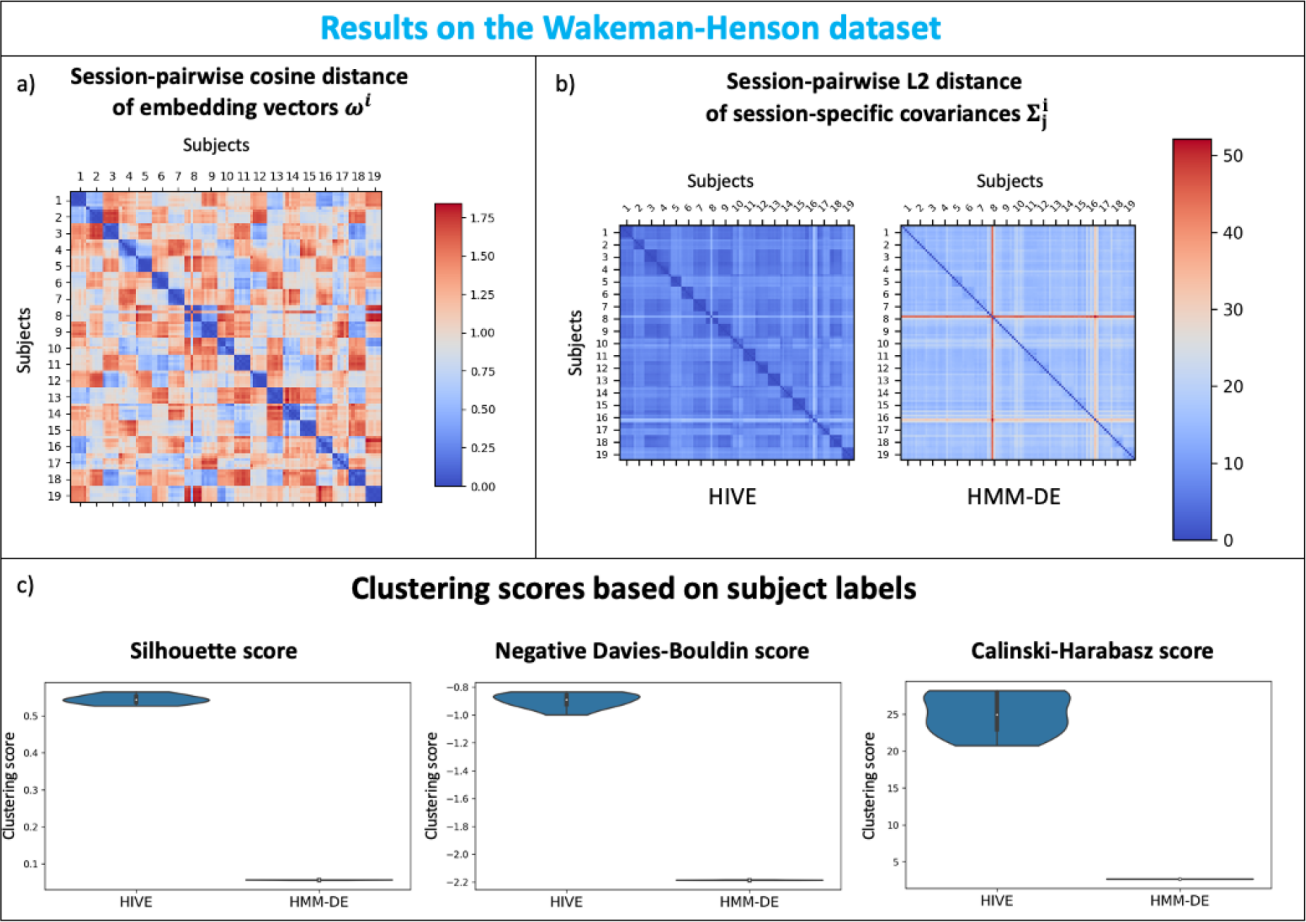
Wakeman Henson: HIVE reveals similarities and differences between MEG recordings. (a) Session-pairwise cosine distance of inferred embedding vectors. Embedding vectors for the same subject are grouped together and have smaller distance between them. (b) Session-pairwise L2 distance of inferred covariances from HIVE (left) and HMM-DE (right). (c) Clustering metrics - Silhouette score (left), negative Davies-Bouldin score (middle), Calinski-Harabasz score (right), based on subject labels for 10 independent runs of both approaches. Higher values for these metrics indicate better clustering.

In this section, we study the Wakeman-Henson dataset (see Section 2.6.2). This dataset contains 6 sessions for each of the 19 subjects, totalling 114 recording sessions. Ideally, we would expect the sessions for a subject to be more similar than sessions for different subjects. In this study, we assign each session an embedding vector and train HIVE on these data with $n_{\omega }=10$ (see SI Section A.1.7). The session-pairwise cosine distances of embedding vectors are plotted in Figure 7a, which shows clear block diagonal structure–session embedding vectors from the same subject are closer together than those from different subjects. This shows the model is able to identify certain recordings which have similar deviations from the group, despite HIVE being trained in an unsupervised manner with no knowledge of which sessions belong to which subjects.

To compare with the traditional approaches, we train both HIVE and HMM-DE on this dataset. In Figure 7b, the session-pairwise L2 distances of inferred covariances from both approaches are plotted. Although the same block diagonal structure can be seen in both approaches, it is clearer in the case of HIVE. One can observe there is a particular session (session 3 of subject 8) that has much higher distance with all other sessions, especially with HMM-DE. This is related to the fact that this particular session has very different oscillatory activity compared to other sessions (see SI Section A.1.8). In order to quantify the advantages with HIVE, we employ 3 different metrics—Silhouette score (Rousseeuw, 1987), Davies-Bouldin score (Davies & Bouldin, 1979), and Calinski-Harabasz score (Caliński & Harabasz, 1974) for assessing how well the inferred covariances form distinct and well-separated subject clusters. Figure 7c shows that with all three metrics, HIVE inferred covariances form tighter and more distinct clusters than dual estimated covariances from HMM-DE.

#### 3.2.2 Combined dataset: HIVE reveals systematic variability across different scanners

**Fig. 8.**
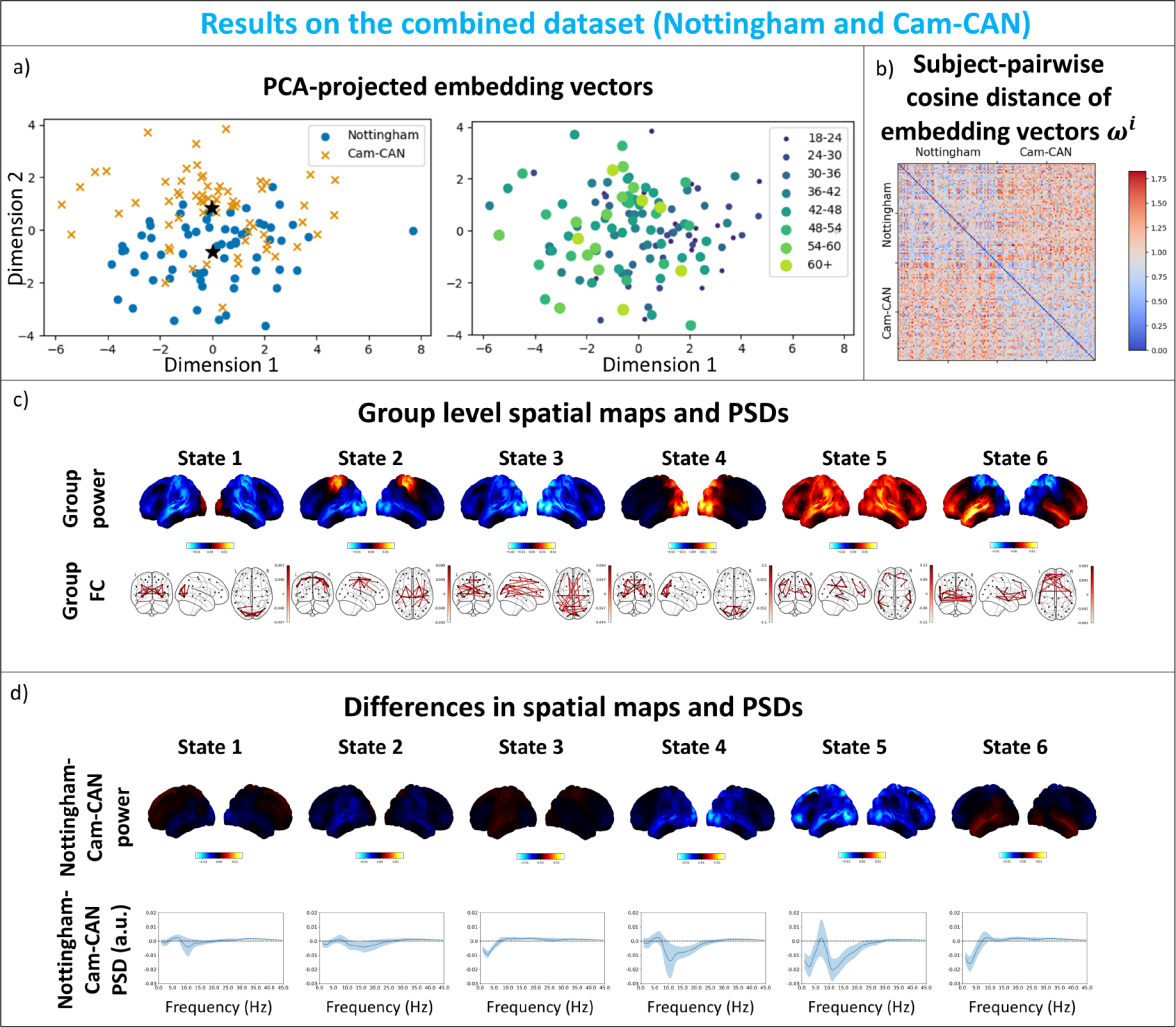
Combined dataset: HIVE reveals systematic variability across different scanners. (a) PCA-projected embedding vectors show axes that reflect site and age. They are coloured and marked with different datasets (left, black stars indicate centroids of embedding vectors from the two datasets) and different age groups (right, older subjects are coloured with lighter colours and larger dots). (b) Inter-subject differences reflect site differences. Subject-pairwise cosine distance of embedding vectors. (c) Group-level power maps and networks. 6 states are inferred with HIVE. The top and bottom rows show the group-level power (red areas show above average and blue areas show below average power across states) and FC maps (top 3% edges are plotted). (d) Differences between sites in power. The top row shows power difference (red areas show higher and blue areas show lower power in Nottingham subjects than Cam-CAN subjects) for each state between centroids of both datasets. The bottom row shows the difference in PSDs (solid lines show the means, and shaded areas show one standard deviation across the parcels) across frequencies between datasets.

In this study, we want to test if our model is able to differentiate data acquired by different scanners. We train on two different resting-state MEG datasets (Nottingham and Cam-CAN) described in Section 2.6.2. There are 128 subjects in total and 64 subjects from each dataset. To avoid the possibility that the model is biased towards either of the datasets, we match the age and sex profiles. Here, we choose $n_{\omega }=20$ (see SI Section A.1.7). We can clearly see two clusters of embedding vectors inferred in Figure 8a, and at the same time an age gradient in the embedding space. This means scanner type and age information are simultaneously encoded (in different directions in the embedding space) by the embedding vectors, despite the fact that HIVE is trained unsupervised with no knowledge that the sessions were from different scanner types. We can also see a block diagonal structure in Figure 8b where recording sessions scanned by the same scanner have smaller pairwise cosine distances of their inferred embedding vectors. With the clustering metrics described in Section 3.2.1, we see from Figure A10 that subject-specific covariances from HIVE form better-defined clusters based on scanners/sites.

HIVE also provides a way to summarise the differences in state-specific spectral content between scanner types. For both of the datasets, the centroids of the embedding vectors are computed (shown as black stars in the left panel of Fig. 8a). For each of the centroids, we select ten nearest neighbours in the embedding space, whose spectral content are averaged to give a representation of spectral contents of each of the datasets. In Figure 8d, we can see these differences between the “centroids” of the datasets. In particular, subjects from the Nottingham dataset generally have lower power than those from Cam-CAN, especially in posterior regions and alpha band.

#### 3.2.3 Cam-CAN: HIVE reveals individual variability across age

**Fig. 9.**
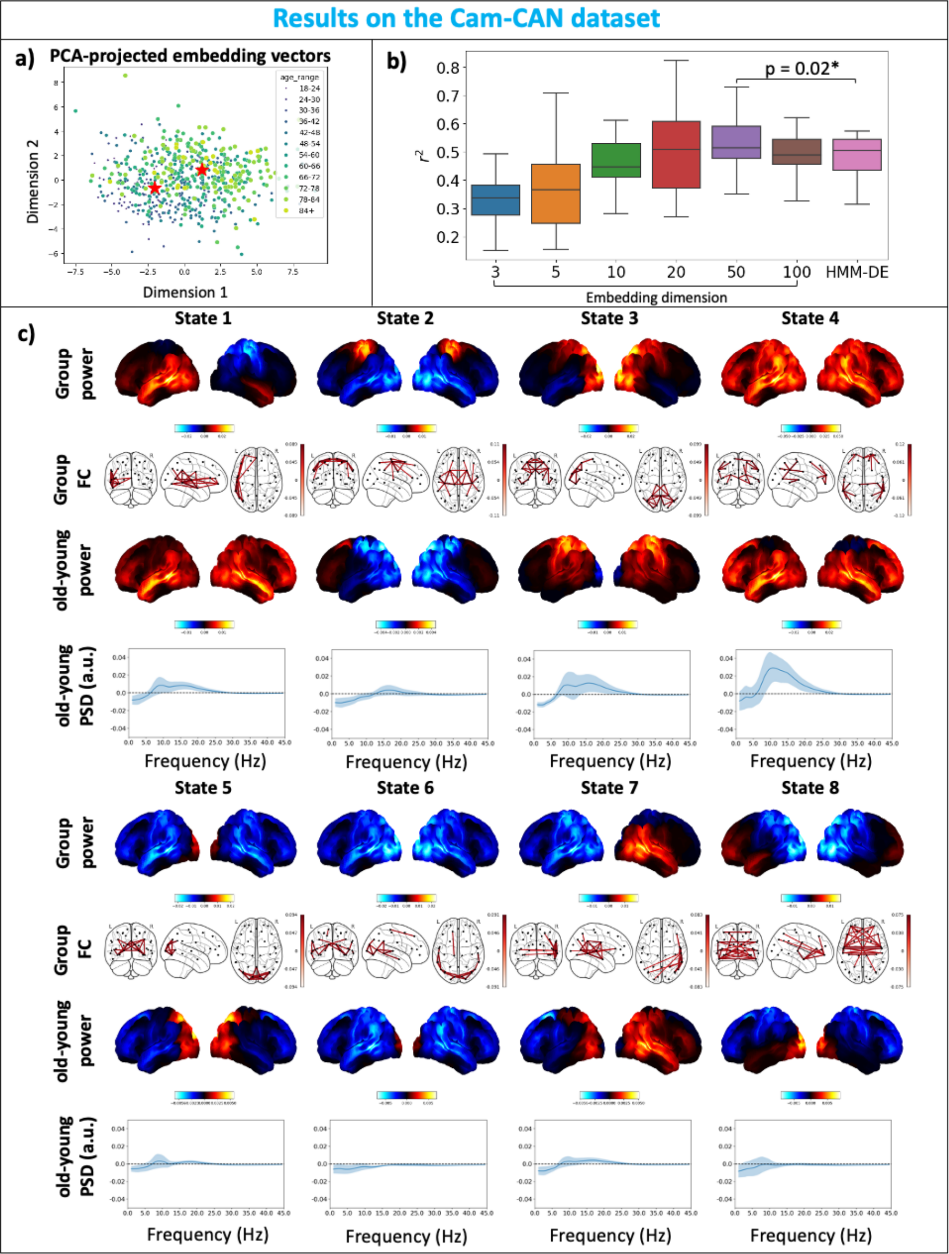
Cam-CAN: HIVE reveals individual variability across age. (a) Inferred embedding vectors projected to 2 dimensions with PCA. Darker-coloured and smaller dots are younger participants. Lighter-coloured and larger dots are older participants. The two red stars show centroids of subjects with age <36 and >72 . (b) 20-fold cross-validated age prediction accuracy for HIVE with different embedding dimensions and HMM-DE. (c) 8 states are inferred by HIVE. Group-level power (red areas show above average and blue areas show below average power across states) and FC maps (top 3% edges are plotted) are shown as well as the differences in power (red areas show higher and blue areas show lower power in older subjects than younger subjects) and PSDs (solid lines show the means, and shaded areas show one standard deviation across the parcels) between old and young subjects.

Here, we use the Cam-CAN dataset described in Section 2.6.2, that consists of 612 healthy subjects aged between 18–88 and choose $n_{\omega }=50$ (see SI Section A.1.7). From Figure 9a, we can clearly see an age gradient from darker, smaller to lighter, larger dots. This means after training, age information is encoded by the embedding vectors of the subjects, despite the fact that HIVE is trained unsupervised with no knowledge of the subjects’ ages. In order to see if the learnt hidden representation given by the embedding vectors helps improve the model’s ability to distinguish between subject demographics, we try to predict age with the inferred subject-specific covariances. Here, HMM-DE and HIVE with increasing embedding dimensions are trained on this dataset. Shown in Figure 9b are the distributions of accuracy (given by different folds of cross-validation) of predicting age with inferred subject-specific covariances (see SI Section A.1.9 for details). The coefficient of determinant ($r^{2}$) is used as a measure of prediction accuracy and it gradually increases with the embedding dimension until there is a significant improvement over HMM-DE when using an embedding dimension of 50. There is a slight drop in accuracy when using an embedding dimension of 100, which could be due to overfitting. Notice that when selecting the embedding dimension, we do not see a significant decrease in variational free energy if we increase $n_{\omega }$ from 50 to 100 (Fig. A3).

Centroids in the embedding space of subjects of age groups <36 and >72 are used as representatives of young and old subjects. Similar to the analysis in Section 3.2.2, we can find the nearest neighbours (here we choose 20 subjects) for both representatives and get the power maps, PSDs for young and old subjects. We show the results in Figure 9c. We can conclude that HIVE can discover meaningful age patterns in the data.

## 4 Discussion

In this paper, we propose the use of embedding vectors as a means of characterising functional networks in different sessions/subjects, similar to how word embedding vectors characterise semantic differences between words in a dictionary. These embedding vectors are incorporated into a generative model of FC that uses covariance matrices to describe network activity. This can be potentially used in many different variants of brain network models, including static (time-averaged) approaches and other dynamic network models (e.g., DyNeMo, Gohil et al., 2022).

In HIVE, similar to the approach taken in PROFUMO (Harrison et al., 2015, 2020), session deviations from the group-level estimates are generated through a Bayesian prior. The additional feature of embedding vectors allows the model to find subpopulations in the group. By training HIVE on the Wakeman-Henson dataset, we observe that inter-subject variability is much greater than inter-session variability (Section 3.2.1). This was also found in fMRI literature (Gratton et al., 2018).

There might be concern over potential over-regularising the variability by the exponential prior on deviation magnitude based on results shown with Simulation 2 (Fig. 5c) and with the Wakeman-Henson dataset (3.4b). However, the amount of regularisation provided by the exponential prior depends on the learnable MLP decoder and embedding vectors, that is, the model can learn to automatically adjust the amount of regularisation through training. We believe the shrinkage effect when there is not enough data and excess of noise is the preferred behaviour. Results in Sections 3.2.1 and 3.2.2 that show HIVE inferred covariances form better-separated clusters with either subject labels or scanner types than dual estimated covariances, as well as the fact that HIVE inferred covariances have greater prediction power of age over dual estimated covariances (Fig. 9b), should help mitigate this concern. Readers might also be concerned about the performance of HIVE trained with different data quality; we have provided the results of training HMM-DE and HIVE on simulated data with different signal-to-noise ratio and it shows HIVE can perform very well with SNR as low as 0.5 (SI Section A.1.13).

Using simulations, we show that during inference the variability encoding block learns multivariate session-specific deviations (Section 3.1.1). This is an important feature because multivariate session-specific deviations are expected in real data and this model is capable of learning these. Additionally, we see that the model can accurately infer the pairwise relationships between sessions, that is, discover subpopulation structure through the embedding vectors. We also demonstrated this through three real data studies where we show the structure in the space of embedding vectors is a manifestation of subject (Section 3.2.1), scanner-type (Section 3.2.2) and age (Sections 3.2.2, 3.2.3) differences. These results have profound implications in normative modelling. For instance, the embedding vectors of subjects can be seen as reference points in a population, extracted in a data-driven way. The effects of ageing, deviations from the group, disease progression, etc, can be studied in the space of embedding vectors. The embedding vectors might seem to be abstract representations of the data itself and could be hard to interpret. But thanks to how we formulate the generative model, we can generate the networks of any point, including those not in the training set, in the space of embedding vectors by passing the embedding vector through a trained variability encoding block. Another approach, which is taken in this work, is to aggregate networks from the nearest neighbours (Sections 3.2.2, 3.2.3).

Potential application of the proposed framework also extends to data harmonisation. For example, suppose we have data from multiple datasets, potentially scanned with different scanners. We can assign different embedding vectors for recordings from different datasets and the same embedding vectors for recordings from the same dataset. By doing this, we can remove the dataset effect on the group-level estimates of networks and retain variability between recordings from the same dataset. Moreover, the proposed framework provides a natural way to isolate different sources of variation (i.e., different sets of embedding vectors for different sources of variation). In the example above, aside from embedding vectors for different datasets, we can also assign embedding vectors for different age groups, sexes, etc. Hence, the effect of each source of variation can be studied independently in their own space of embedding vectors.

Similarly, apart from differences in data sources, different choices in preprocessing pipeline, source reconstruction/parcellation can affect the individual networks/power maps and it is possible to find different inter-subject similarities/relationships for different preprocessing/source reconstruction/parcellation choices. Studying these effects will be interesting future work and HIVE can provide a good basis for studying the effects of these different combinations of data processing choices.

Transfer learning is also a potential use case of the proposed model. It is often the case that studies for specific demographics or diseases have a small amount of data (we call these “boutique” studies), which could result in a lack of statistical power for discovery. Given the evidence in this work that HIVE inferred networks form better separated clusters (Sections 3.2.1, 3.2.2) and provide more prediction power (Section 3.2.3) than HMM-DE, an important next step for this model would be to train the model on large-scale datasets, and either apply or fine-tune the trained model on the boutique dataset to see if statistical power can be improved.

In this paper, we focused on applying HIVE to MEG data. MEG is an interesting application of HIVE, since, as well as capturing variability over sessions in spatial maps of power, it can also capture variability in the auto and cross-spectra, which have been shown to have strong predictive power (Stier et al., 2025).

However, there is no reason why the proposed model cannot be applied to fMRI data where the number of subjects is much larger, especially given the observation in Figure 6, that HIVE provides significantly more improvement in accuracy compared to HMM-DE with increasing numbers of sessions. However, we acknowledge that the number of samples per subject is much lower in fMRI and the linear trend shown in the left panel of Figure 6 is unlikely to hold if we have an extremely high number of subjects (e.g., in UK Biobank). A comprehensive study on fMRI data is beyond the scope of the current work, but is no doubt, in our opinion, an important direction to explore.

In Section 2.3, we mentioned that we assume all sessions share the same transition probability matrix. For our experiments we found as long as we have enough data per subject, this assumption has a tiny effect on the results. After all, the transition probability matrix is part of the prior distribution and will be overridden by the likelihood when there is enough data. However, a formal investigation is needed when the model is applied to datasets where the amount of data per session is relatively small, for example, as is typically the case in fMRI.

It should be noted that if we choose to colour the PCA-projected embedding vectors in Figure 9a with, for instance, sex, we cannot see clear clustering according to sex (Fig. A6). The reason for this is two-fold. Firstly, PCA is used to visualise embedding vectors, which means only the two directions of biggest variation of the embedding vectors are visualised. This is the reason why we chose to present differences in age and scanner-types. However, we could use techniques like Linear Discriminant Analysis (LDA, Fisher, 1936) to find the linear transformation of the session embedding vectors that best separates a specific source of variation. Secondly, during optimisation of the loss function in Equation (13), it might not be as rewarding to group embedding vectors according to sources of variation that have a smaller effect than those that have a larger effect on the variability of the data. To solve this, a potential solution will be to assign embedding vectors for different sources of variation so that the effects of each source of variation are separated.

Due to the additional complexity of the model, HIVE has more hyper-parameters than the HMM, including the number of layers, number of neurons per layer in the decoder of the variability encoding block. However, in practice, we find that results are very robust to the choice of these hyper-parameters and we have used the same set of hyper-parameters for all simulation and real data studies (see SI Section A.1.6). We suggest users of this model to use the same set of hyper-parameters used in this paper. Furthermore, HIVE is remarkably stable over independent runs of different initialisations of model parameters. We show this in SI Section A.1.12. Although we have shown in Figure 9b that prediction performance changes with the number of embedding dimensions, the conclusion that an age gradient can be observed in the embedding space is consistently found across a wide range of choices for the number of embedding dimensions, and the same can be said about the differences in scanner types shown in Figure 8a. Moreover, when we do not see a significant decrease in the variational free energy when increasing the embedding dimension, we see a drop of prediction power of age with HIVE inferred covariances (from $n_{\omega }=50$ to $n_{\omega }=100$ , see Fig. 9b and Fig. A3). Nevertheless, we acknowledge that finding a principled way that does not depend on heuristics or post-hoc analysis is an important future direction to explore.

Choosing the number of states in the state-based models, including HMM and HIVE, is a long standing challenge. This work does not attempt to address this issue and we make sure all the conclusions we made in this paper are independent of the choice of number of states. Previous studies have shown the variational free energy keeps decreasing with increasing number of states (Baker et al., 2014), similarly for other dynamic network models (Gohil et al., 2022). We hypothesise that this could be due to the inter-session variability in the data (see Fig. A5) and modelling variability in the data could be a potential solution to this problem. In this work, we have provided a proof of concept on the possibility of modelling variability with embedding vectors. However, this body of work is by no means perfect. Future work is needed to validate the model further.

## 5 Conclusion

We proposed the use of embedding vectors to model individual functional neuroimaging sessions and applied this approach to extend the HMM, giving us HIVE. The variability encoding block explicitly models variability within a population in a principled way. We provide a way to perform efficient inference on the model parameters and the algorithm is readily scalable to large amount of data. With a Bayesian prior, the model pools information across individuals for how they may deviate from the group mean. The embedding vectors allow the model to group together similar data and help the interpretation of sources of variation in a population. This is an important step towards making use of the numerous large-scale datasets collected with different protocols. We believe the above results demonstrate that the proposed model provides a novel perspective in population modelling and in the inference of functional networks.

## Supplementary Material

Supplementary Material

## Data Availability

Data used are publicly available. Availability of the Nottingham dataset is at the official MEGUK site: https://meguk.ac.uk/database/. For the Wakeman-Henson dataset, we refer the readers to the original paper (Wakeman & Henson, 2015). For the Cam-CAN dataset, we refer the readers to the original paper (J. R. Taylor et al., 2017). Source code for HIVE is available in the *osl-dynamics* toolbox (Gohil et al., 2023) and scripts to reproduce results in this paper are available here: github.com/OHBA-analysis/Huang2025_ModelVariabilityWithEmbeddings.
