# Supplementary material for: Modelling variability in functional brain networks using embeddings
Source: Imaging Neurosci (Camb). 2026 Apr 17;4:IMAG.a.1188. doi: 10.1162/IMAG.a.1188 (PMC13094015; doi:10.1162/IMAG.a.1188)
Supplement: Supplementary Material [file IMAG.a.1188_supp.pdf]

A.1 Supplementary Information (SI)

A.1.1 Generative model of the HMM

In the HMM, data from different sessions are concatenated temporally. Let $\boldsymbol{x}_t$ be the observed data at time $t \in [T]$, where T is the total number of time points and $\boldsymbol{x}_t$ is a vector of length N_c - the number of channels/parcels. Furthermore, let s_t be the hidden state at time t, which follows a discrete Markov process. It is assumed in the HMM that the data is generated by

$$\boldsymbol{x}_t | (s_t = j) \sim \mathcal{N}(0, \Sigma_j) \quad \forall t \in [T], \tag{1}$$

independently. Here Σ_j is a $N_c \times N_c$ covariance matrix when state j is active.

A.1.2 Dual estimation of HMM

For session each i, let γ^i_{jt} be the state probability of state j at time t, then the session-specific covariances $\{\boldsymbol{\Sigma}^i_j\}_{j=1}^J$ are estimated by

$$\hat{\boldsymbol{\Sigma}}^i_j = \frac{\sum_{t=1}^{T_i} \gamma^i_{jt} (\boldsymbol{x}^i_t - \bar{\boldsymbol{x}}^i)(\boldsymbol{x}_t - \bar{\boldsymbol{x}}^i)^T}{\sum_{t=1}^{T_i} \gamma^i_{jt}} \tag{2}$$

A.1.3 Flowchart for HIVE training loss

See Figure A1.

A.1.4 KL annealing

Optimising the parameters of a model with a powerful decoder is challenging. In most runs, the **KL** term dominates and the model consistently sets the variational distribution to be close to the prior distribution of the latent variables, yielding a very small **KL** term (Bowman et al., 2015). The solution to this is surprisingly simple and effective: annealing the **KL** term. The idea is to train the model with a modified loss function where the **KL** term is multiplied by an annealing factor κ that starts at 0 and gradually increases to 1 as the training progresses. In our case, the modified loss is

$$\mathcal{L}_n(\boldsymbol{\phi}, \boldsymbol{\psi}) = -\mathbf{LL}_n(\boldsymbol{\phi}, \boldsymbol{\psi}) + \kappa \mathbf{KL}_n(\boldsymbol{\phi}, \boldsymbol{\psi}) \tag{3}$$

This allows the model to first focus on reconstructing the observed data before tuning the variational distribution to match the prior distribution. In this paper, we use a tanh scheme for the annealing factor κ:

$$\kappa = \begin{cases} 0 & \text{if } epoch = 1 \\ 1 & \text{if } epoch \geq N_{anneal} \\ \frac{1}{2} \tanh\left(sharpness \frac{[(epoch-1) \mod N_{anneal}] - N_{anneal}/2}{N_{anneal}} \right) + \frac{1}{2} & \text{otherwise,} \end{cases} \tag{4}$$

where N_{anneal} is the number of annealing epochs beyond which $\kappa = 1$ and *sharpness* is a hyper-parameter that controls the steepness of how κ increases. The trick of annealing the **KL** term during training involves tuning many hyperparameters. Recently there have been more principled ways for training VAEs (e.g. Rezende and Viola, 2018), and these methods can be explored in the future for better training of the model.

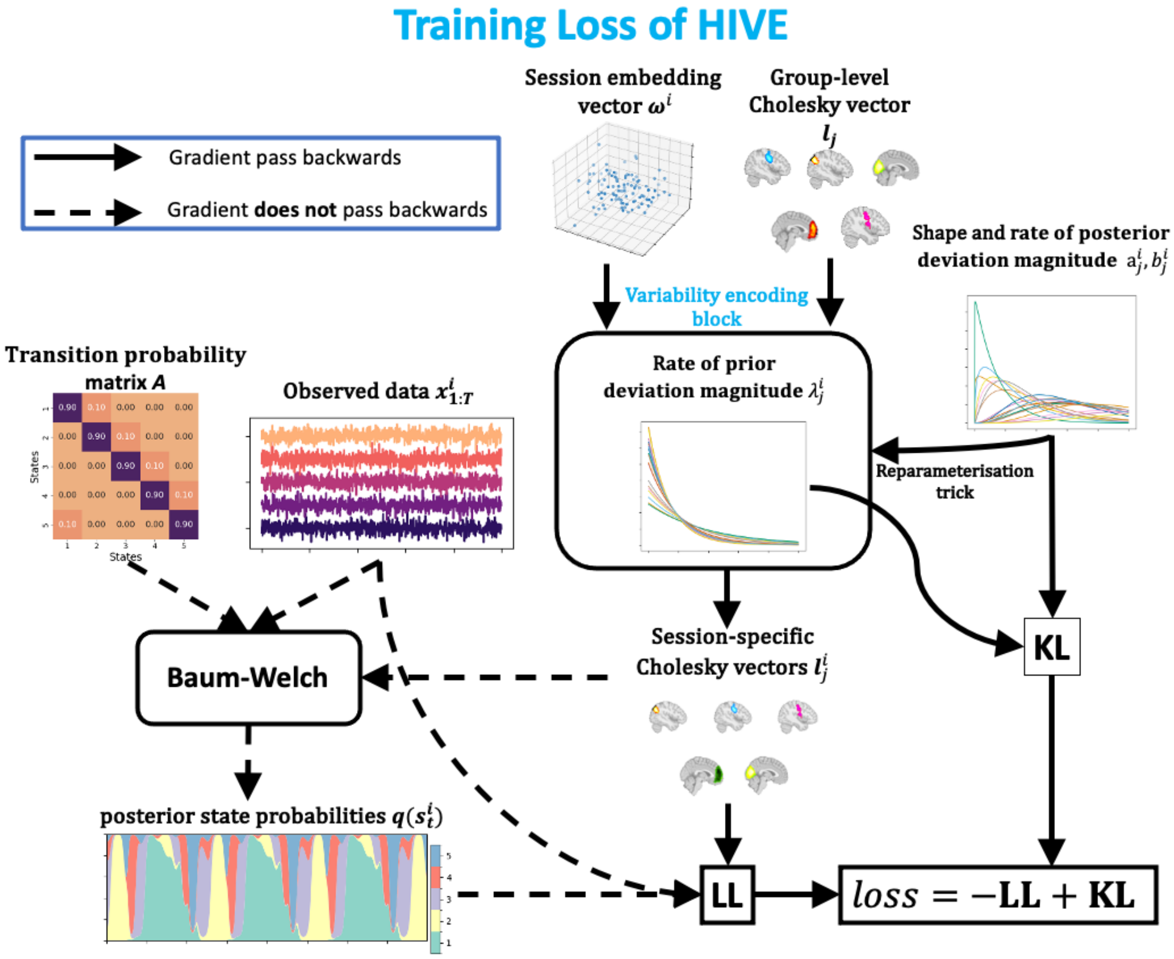

Figure A1: **The full HIVE training framework**. The black solid arrows in the plot show connections where gradient flows through during back-propagation and the dashed black arrows show connections where gradient does not flow through.

A.1.5 Data simulation

In this paper, data simulation involves three steps:

- Simulating the underlying hidden state time courses of the brain dynamics.
- Simulating covariances.
- Simulating the observed data given the hidden state time courses and covariances.

A.1.5.1 Simulating the hidden state time courses

Binary time courses can be generated with a Markov chain, i.e. given the number of states J, the transition probability matrix $\boldsymbol{A}$, we generate the hidden time course $s_{1:T}$ with

$$s_t | s_{t-1} \sim \mathrm{Cat}(\boldsymbol{A}_{s_{t-1},:}) \quad \text{for } t = 2, \ldots, T, \tag{5}$$

where s_1 is fixed to be the first state.

A.1.5.2 Simulating group-level covariances

To simulate random covariances, we first sample a $N_c \times N_c$ matrix W_j whose entries are sampled independently from $\mathcal{N}(0, 0.1)$. Then to replicate the case where we have activations in different channels, a large value is added to randomly selected rows of W_j. Then we set $\boldsymbol{\Sigma}_j = W_j W_j^T + \epsilon \boldsymbol{I}$, where $\epsilon = 10^{-6}$ is added to the diagonal to ensure the matrix is positive definite.

A.1.5.3 Simulating session-specific covariances

In the case where we want to simulate session-specific covariances, we first generate group-level covariances as described in Section A.1.5.2. Each session is associated with an embedding vector, which is assigned a group uniformly randomly from a set of groups. Each embedding vector is generated with a multivariate Gaussian distribution with the same variance but different means depending on the group assignment. Next linear transformations are generated randomly to map the group-level Cholesky vectors to spatial embeddings for each state/mode, which are concatenated with the embedding vectors to give the concatenated embeddings. The session-specific deviations are generated by applying another randomly generated linear transformation to the concatenated embeddings. Finally, the session-specific covariance matrices are generated by adding the session-specific deviations to the group-level covariances.

In Sections 3.1.2, 3.1.3 and Appendix Section A.1.13, 3 groups of sessions are simulated and we show the mean deviation from the group covariances of each of the groups in Appendix Figure A2.

A.1.6 Model specifications

Here we present the model specifications of HMM in Tables A1, A2 and of HIVE in Tables A3, A4 when training on different datasets.

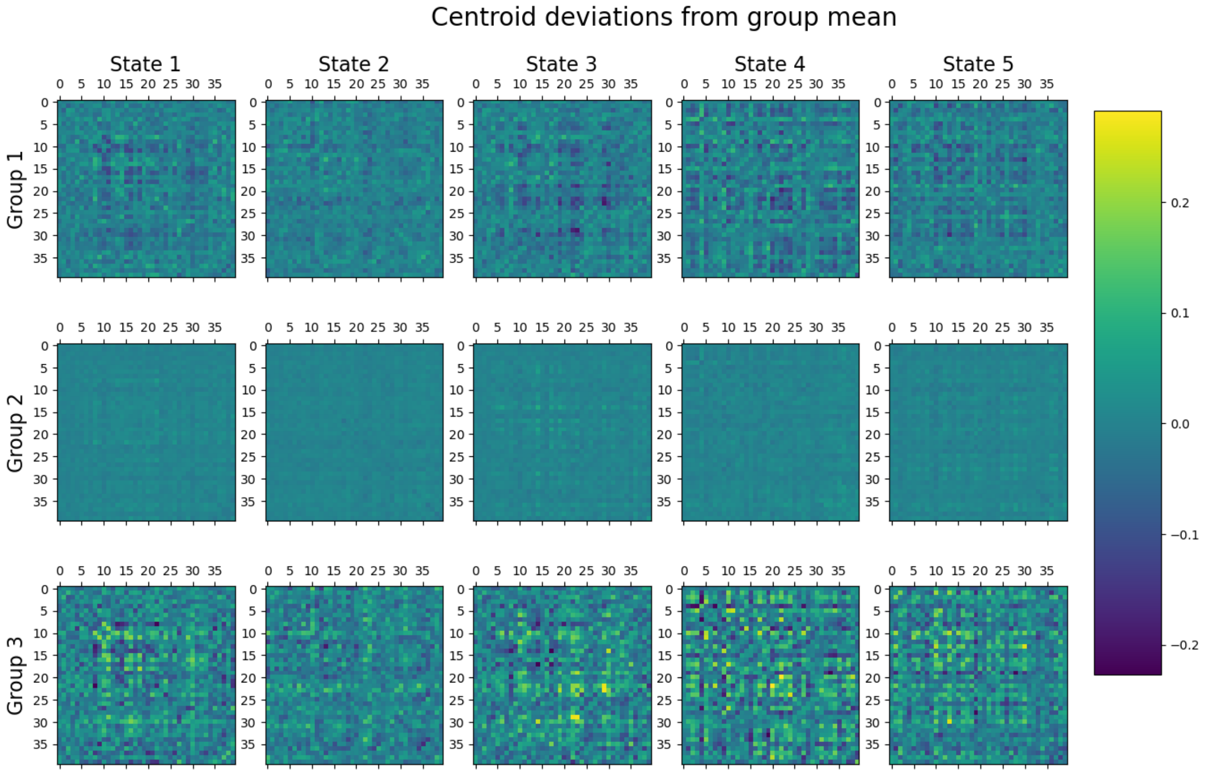

Figure A2: **Centroid deviations of 3 groups of session-specific covariances.** Each row show the mean deviation of session-specific covariances in each group from the group-level covariances.

Hyper-parameters	Simulation 1	Simulation 2	Simulation 3
Number of states	5	5	5
Sequence length	200	200	200
Training epochs	40	40	40
Batch size	32	64	32
Learning rate	1e-2	5e-3	5e-3
Learning rate decay factor	0.05	0.05	0.05
Number of multi-starts	10	5	5
Multi-start epochs	5	3	5
Number of parameters	330	4,100	4,100

Table A1: Model specifications for training HMM on simulated datasets.

Hyper-parameters	Wakeman-Henson	Combined dataset	Cam-CAN
Number of states	6	6	8
Sequence length	2000	2000	2000
Training epochs	20	20	10
Batch size	32	32	32
Learning rate	1e-2	1e-2	1e-3
Learning rate decay factor	0.1	0.1	0.1
Number of multi-starts	3	3	3
Multi-start epochs	1	1	1
Number of parameters	19,440	19,440	25,920

Table A2: Model specifications for training HMM on real datasets.

Hyper-parameters	Simulation 1	Simulation 2	Simulation 3
Number of states	5	5	5
Sequence length	200	200	200
Embedding dimension	2	10	10
Spatial embedding dimension	2	2	2
Decoder number of layers	5	5	5
Decoder number of units	32	32	32
Decoder activation	tanh	tanh	tanh
Decoder regularisation	L1	L1	L1
Decoder regularisation strength	10	10	10
Annealing sharpness	10	10	10
Annealing epochs	20	20	20
Training epochs	40	40	40
Batch size	32	128	32
Learning rate	5e-3	5e-3	5e-3
Learning rate decay factor	0.05	0.05	0.05
Number of multi-starts	10	10	5
Multi-start epochs	5	5	5
Number of parameters	7,590	40,640	38,840 - 40,640

Table A3: Model specifications for training HIVE on simulated datasets.

Hyper-parameters	Wakeman-Henson	Combined dataset	Cam-CAN
Number of states	6	6	8
Sequence length	200	200	200
Embedding dimension	10	20	50
Spatial embedding dimension	2	2	2
Decoder number of layers	5	5	5
Decoder number of units	32	32	32
Decoder activation	tanh	tanh	tanh
Decoder regularisation	L1	L1	L1
Decoder regularisation strength	10	10	10
Annealing sharpness	10	10	10
Annealing epochs	15	20	15
Training epochs	30	40	30
Batch size	128	128	128
Learning rate	5e-3	5e-3	2.5e-3
Learning rate decay factor	0.1	0.1	0.1
Number of multi-starts	10	10	10
Multi-start epochs	2	2	1
Number of parameters	143,619	145,527	189,291

Table A4: Model specifications for training HIVE on real datasets.

A.1.7 Choosing the embedding dimension on the real datasets

We choose n_ω with the procedure described in Section 2.5. For the model trained on the Wakeman-Henson dataset in Section 3.2.1, the decrease in variational free energy is insignificant when comparing $n_\omega = 10$ and $n_\omega = 20$. Hence for this dataset, we choose $n_\omega = 10$. For the model trained on the combined dataset in Section 3.2.2, the tests are all significant and we choose $n_\omega = 20$. For the model trained on the Cam-CAN dataset in Section 3.2.3, the tests are all significant until $n_\omega = 50$. Hence we choose $n_\omega = 50$.

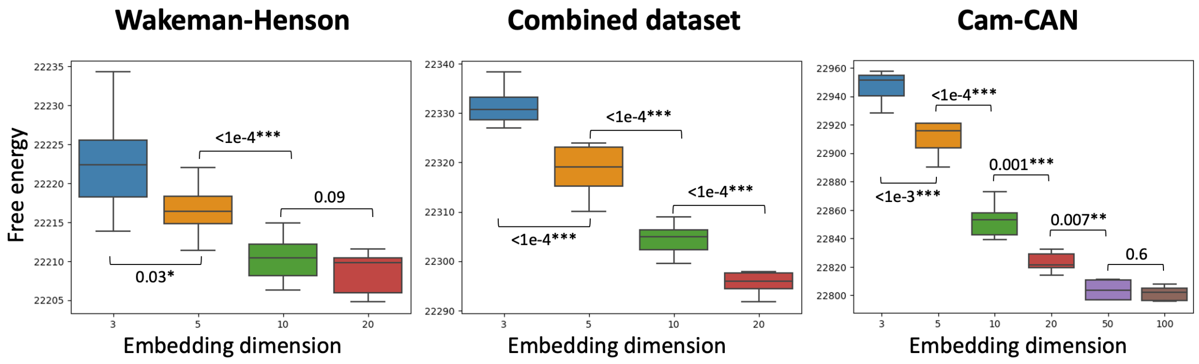

Figure A3: **Free energy is used to choose n_ω.** For each of the real dataset results, different models with different candidate embedding dimensions are trained 10 times independently. The variational free energy on training data is plotted against embedding dimension. The p-values of the progressive t-tests are plotted between the boxplots.

A.1.8 Outlier in the dataset

We see from Figure A4 that run 3 of subject 8 has a very different static PSD profile from the other runs. In particular the spike at around 26 Hz. Furthermore it has a very different session-pairwise correlation of the static covariances from the other runs.

A.1.9 Classifier for predicting age with subject-specific covariances

HMM-DE and HIVE with embedding dimensions $3, 5, 10, 20, 50, 100$ are trained on the Cam-CAN dataset. Each of the 7 models is trained 10 times independently and the best model is chosen based on variational free energy. Next the inferred subject-specific covariances are projected by PCA, whose first few PCs are used as regressors in a ridge regression to predict subject age. The subject age data are separated into 20 folds. For each fold, a classifer is trained on 19 folds and tested on the remaining 1 fold. During training, a 5-fold cross validated grid search is used to select the best dimension of PCA projection and the regularisation strength of the ridge regression. PCA projection dimension is chosen from $\{5, 20, 50, 100\}$ and regularisation strength is chosen from $\{10^i : i \in \{-5, -4, -3, -2, -1, 0, 1, 2, 3\}\}$.

A.1.10 Inter-session variability in data causes ever-decreasing variational free energy with number of states.

We simulate two datasets, one without variability, one with variability between sessions. The ground truth number of states for both datasets is 4. HMM is trained 5 times independently on both datasets and the variational free energy on training data is plotted in Figure A5. We see that when there is no variability, the variational free energy decreases with increasing number of states until it plateaus at the ground truth number of states. However, when there is variability, the variational free energy keeps decreasing with increasing number of states. This is because the model can keep finding more states to explain the inter session variability in the data.

A.1.11 Sex differences in PCA-projected embeddings

Embedding vectors from the study in Section 3.2.3 are coloured and marked with sex of the participant, shown in Figure A6, and no obvious separation or clustering can be observed.

A.1.12 HIVE is reproducible with different initialisations

Here we show the results of 10 independent runs of HIVE with different random initialisations of model parameters on the combined dataset. In particular, we show the variational free energy of each of the runs. Run 4 has the lowest variational free energy and is referred to as the best run, whose results are shown and analysed in Section 3.2.2. We can see from Figure A7 that the group-level power maps are reproducible over all runs and the mean correlation of power maps with the best run chosen based on variational free energy is high (above 0.99). We also show that the inferred transition probability matrices are stable across runs. Notably, the group-level power maps are computed with a multi-taper

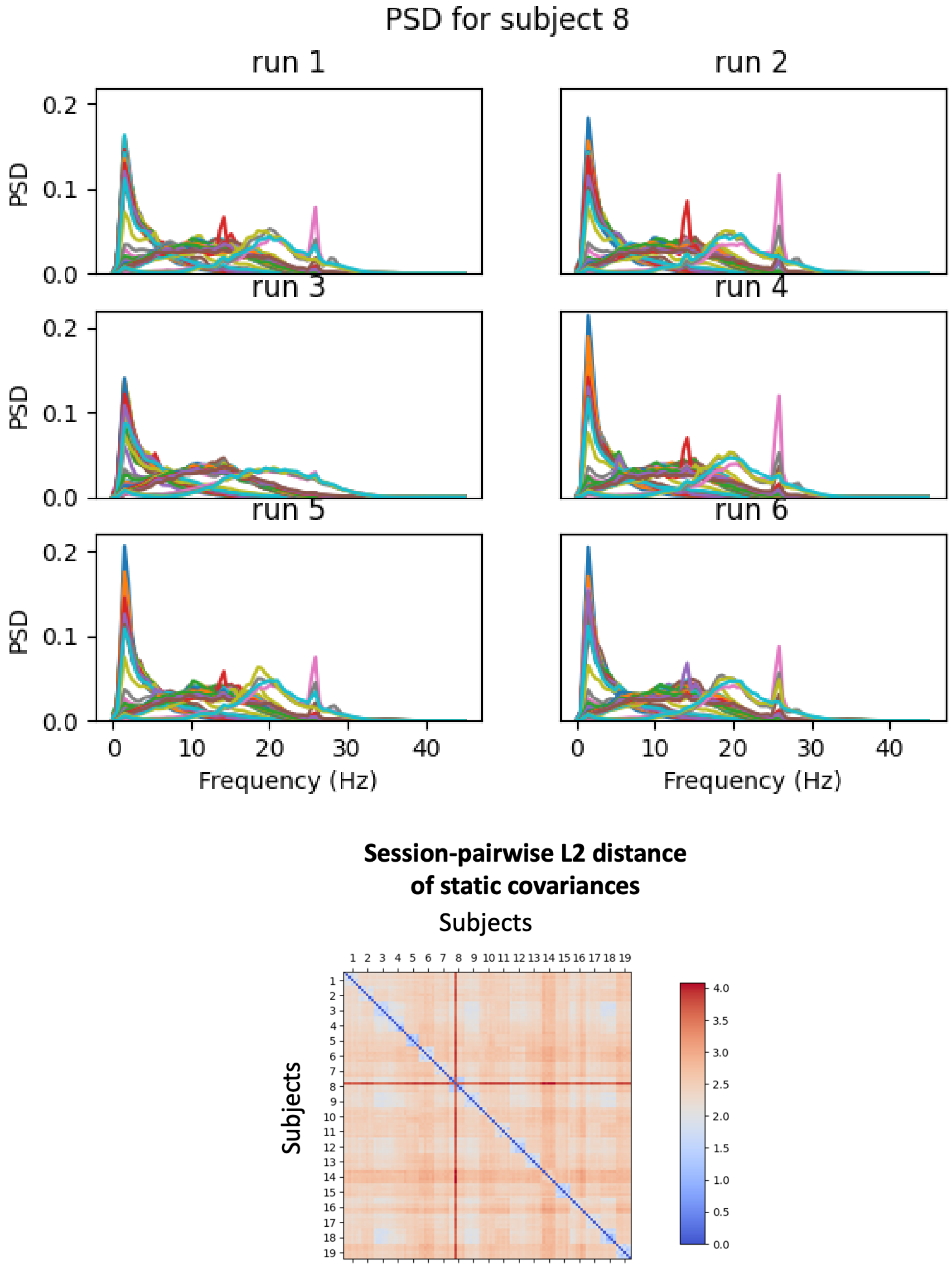

Figure A4: **Outlier in the dataset**. Top: PSD for the prepared data used for training is plotted for different sessions/runs of subject 8. Bottom: The session-pairwise L2 distance of the static covariances is plotted for the prepared data used for training. The outlier run is highlighted in red.

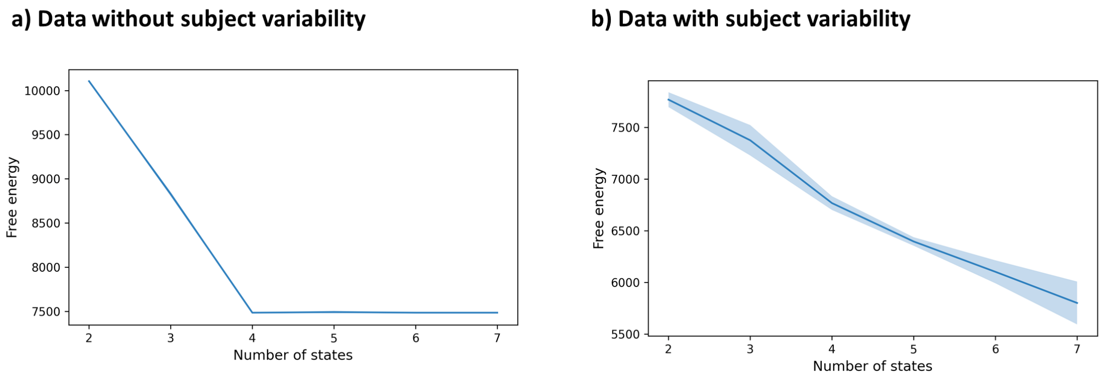

Figure A5: **Inter-session variability in the data can cause ever-decreasing variational free energy in HMM.** a) There is no inter-session variability in the data and variational free energy is plotted against number of states. b) There is inter-session variability in the data and variational free energy is plotted against number of states.

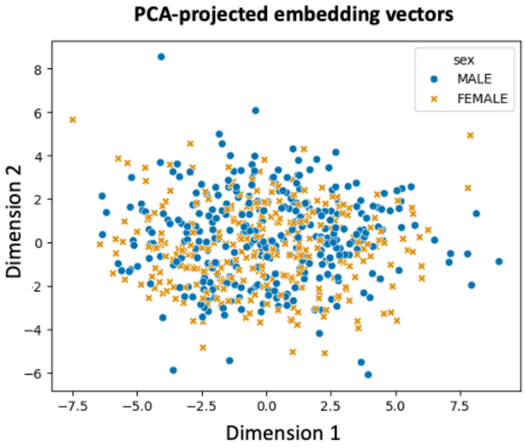

Figure A6: **No visible sex differences in PCA-projected embedding vectors**. *Results obtained using the Cam-CAN dataset.* Inferred embedding vectors projected to 2 dimensions with PCA. Embedding vectors for male participants are plotted as blue dots and those for female participants are plotted as orange crosses.

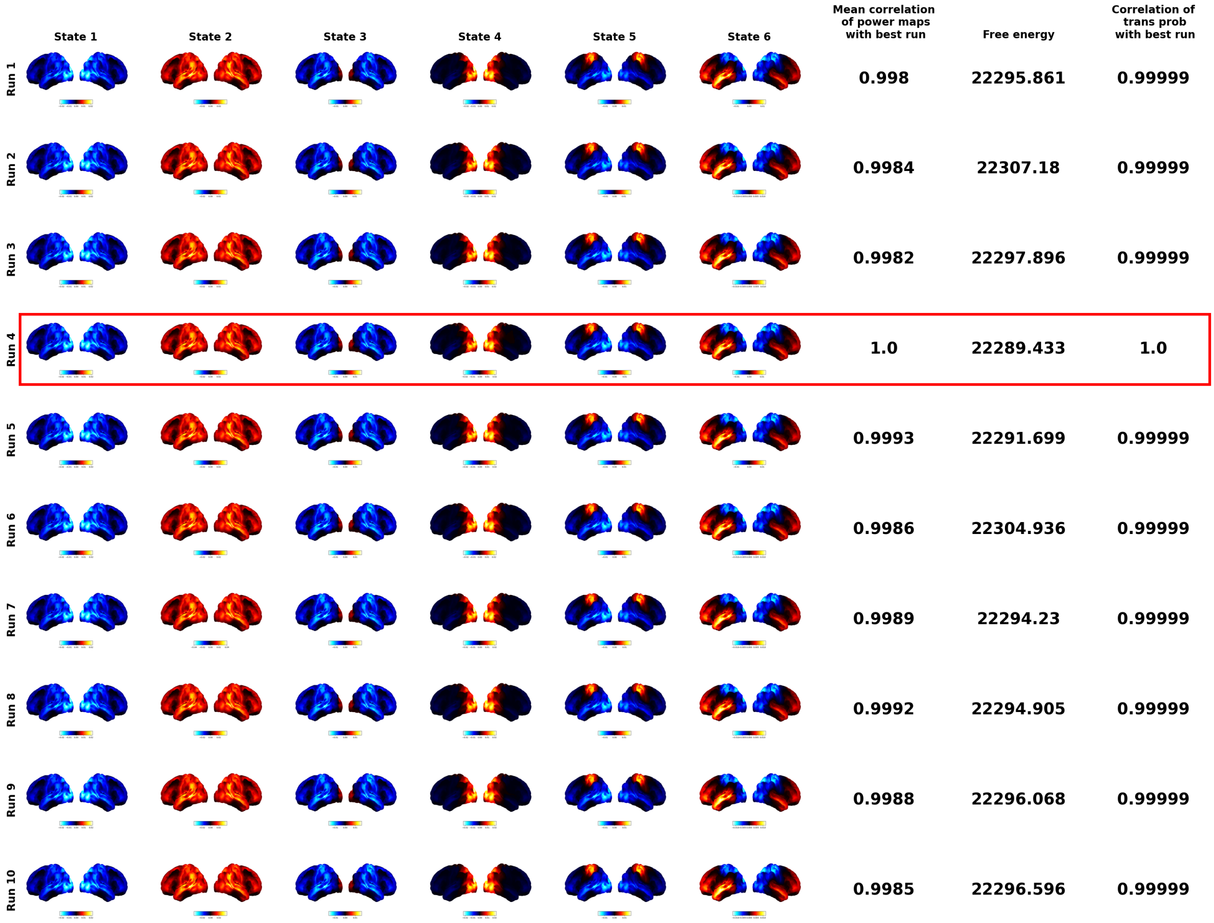

Figure A7: **Combined dataset: HIVE is reproducible with different initialisations of model parameters.** Results of 10 independent runs of HIVE, with different initialisations, trained on the combined dataset are shown. The results include group-level power maps, mean correlation of group-level power maps with the best run, variational free energy, and the correlation of transition probability matrix with the best run. In particular, run 4 is highlighted in red as the best run having the lowest variational free energy. Notice Here the order of states is arbitrary and the states are re-ordered using the Hungarian algorithm to match the power maps.

approach based on the state time courses. Therefore, reproducible power maps will imply similar state time courses.

A.1.13 Performance of HIVE and HMM-DE with varying SNR

Here we simulate datasets of 20 sessions with random session-specific covariances (see Appendix Section A.1.5.3) and add different levels of noise ($\text{SNR} \in \{0.05, 0.1, 0.5, 1, 5, 10, 100, \infty\}$, where $\text{SNR} = \infty$ refers to no noise added) to the resulting simulated data. The final time series data is generated with:

$$\tilde{\boldsymbol{x}}_t^i = \begin{cases} \boldsymbol{x}_t^i & \text{if SNR} = \infty \\ \sqrt{\frac{\text{SNR}}{\text{VAR}}} \times \boldsymbol{x}_t^i + \mathcal{N}(0, \boldsymbol{I}_{N_c}) & \text{otherwise} \end{cases} \tag{6}$$

where VAR is a vector of length N_c, where each element is the variance of each channel of the noise-less data.

In general, we see that HIVE is doing a great job at recovering the ground truth with an SNR above 0.5, even there is little data available, e.g. 1000 samples per session (4s at 250Hz).

A.1.14 Simulation 2 with different variances

Here we repeat the simulation 2 data but with different group variances. In particular, Group 1 has centroid (0, 0) and variance 0.001, Group 2 has centroid (0.15, 0.05) and variance 0.004, Group 3 has centroid (-0.04, 0.06) and variance 0.0001. We can see that the population structure in the embedding space is still recovered by the inferred embeddings.

A.1.15 Extra results on the combined dataset

Here we provide the clustering scores similar to those in Section 3.2.1 applied to the Wakeman-Henson dataset. Here we try to assess the ability of HIVE to separate out sessions from different datasets/scanners. We can see that HIVE consistently provides better separation from the clustering metrics shown in A10.

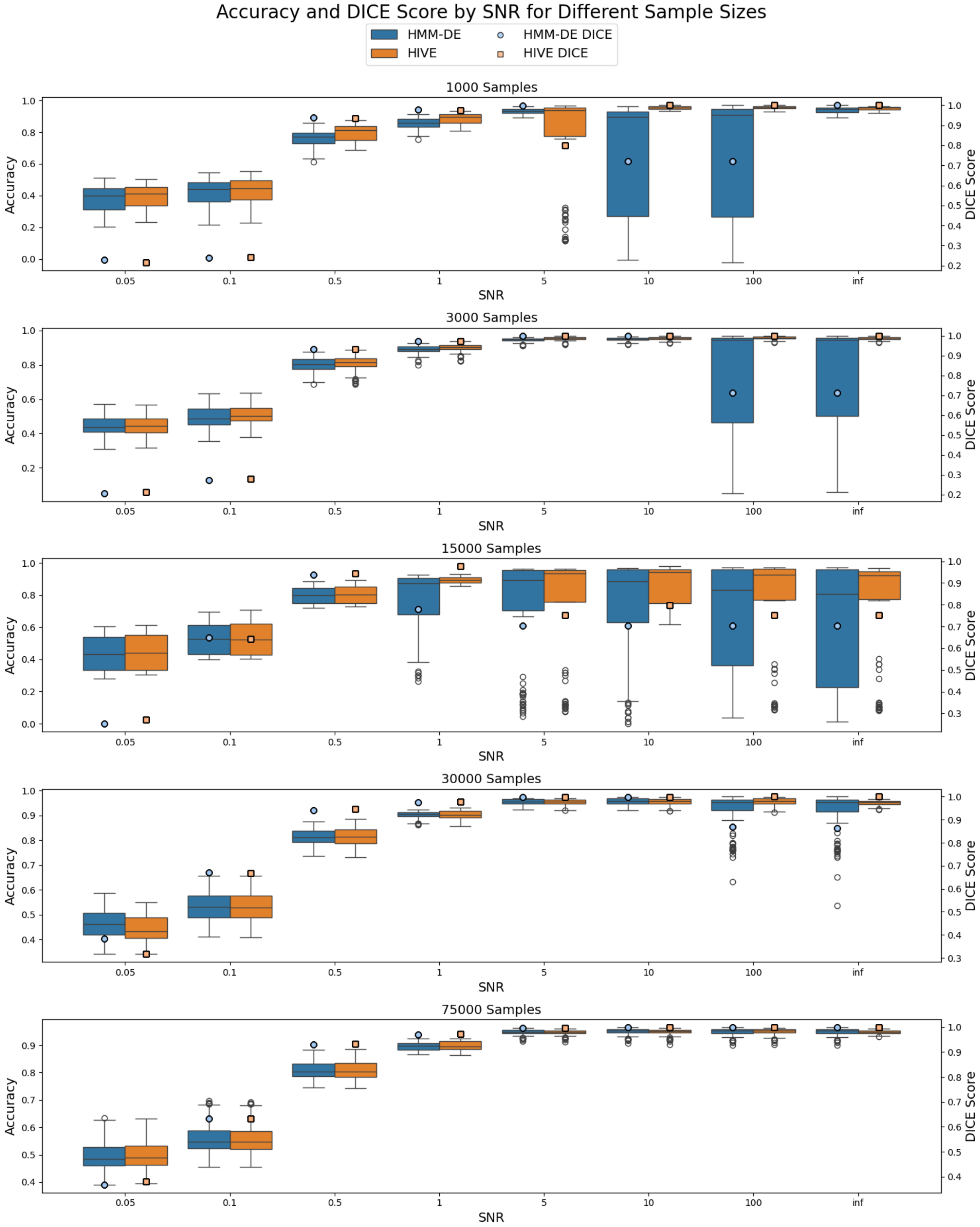

Figure A8: **Simulated dataset: Performance of HIVE and HMM-DE with different SNR and number of samples per session.** Here we show the accuracy (correlation with the ground truth covariances) and DICE score of inferred state time courses from HMM-DE and HIVE over different levels of SNR and number of samples per session.

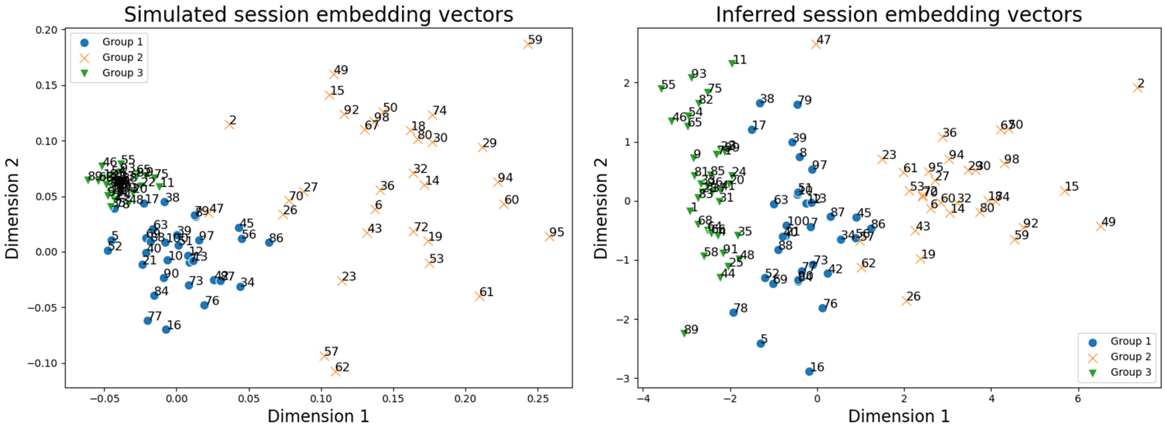

Figure A9: Repeating simulation with different group variances. Group 3 has the smallest variance, followed by Group 1, and group 2 has the largest variance. We can see that the population structure is still recovered.

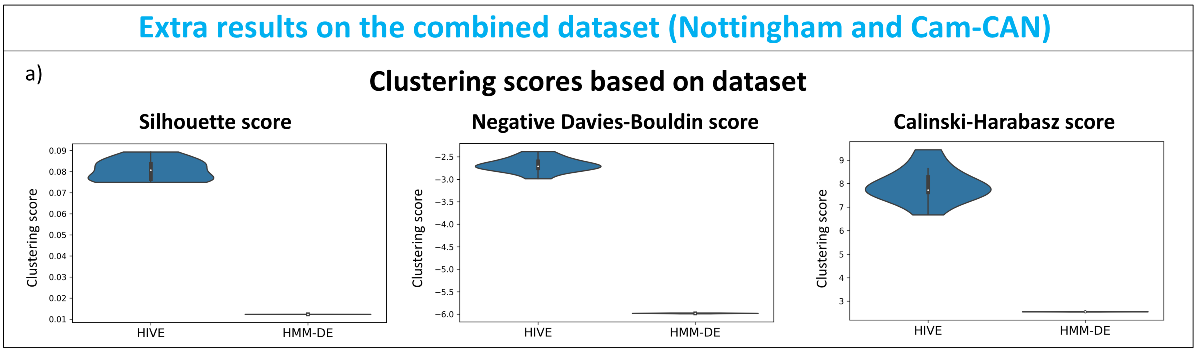

Figure A10: Clustering metrics on combined dataset. Clustering metrics - Silhouette score (left), negative Davies-Bouldin score (middle), Calinski-Harabasz score (right), based on subject labels for 10 independent runs for both approaches.
